# Mitochondrial control of innate immune responses

**DOI:** 10.3389/fimmu.2023.1166214

**Published:** 2023-05-30

**Authors:** Shasha Chen, Zhiyong Liao, Pinglong Xu

**Affiliations:** ^1^ Zhejiang Provincial Key Laboratory for Water Environment and Marine Biological Resources Protection, College of Life and Environmental Science, Wenzhou University, Wenzhou, China; ^2^ Institute of Intelligent Medicine, Hangzhou Global Scientific and Technological Innovation Center, Zhejiang University (HIC-ZJU), Hangzhou, China; ^3^ Ministry of Education (MOE) Laboratory of Biosystems Homeostasis and Protection, Zhejiang Provincial Key Laboratory for Cancer Molecular Cell Biology, Life Sciences Institute, Zhejiang University, Hangzhou, China; ^4^ Cancer Center, Zhejiang University, Hangzhou, China

**Keywords:** mitochondria, innate immunity, mtDNA, mitophagy, mitochondrial metabolism, MAVS, cGAS-STING, inflammasome

## Abstract

Mitochondria are versatile organelles and essential components of numerous biological processes such as energy metabolism, signal transduction, and cell fate determination. In recent years, their critical roles in innate immunity have come to the forefront, highlighting impacts on pathogenic defense, tissue homeostasis, and degenerative diseases. This review offers an in-depth and comprehensive examination of the multifaceted mechanisms underlying the interactions between mitochondria and innate immune responses. We will delve into the roles of healthy mitochondria as platforms for signalosome assembly, the release of mitochondrial components as signaling messengers, and the regulation of signaling *via* mitophagy, particularly to cyclic GMP-AMP synthase-stimulator of interferon genes (cGAS-STING) signaling and inflammasomes. Furthermore, the review will explore the impacts of mitochondrial proteins and metabolites on modulating innate immune responses, the polarization of innate immune cells, and their implications on infectious and inflammatory diseases.

## 1 Overview of mitochondria in innate immunity

**Figure 1:**
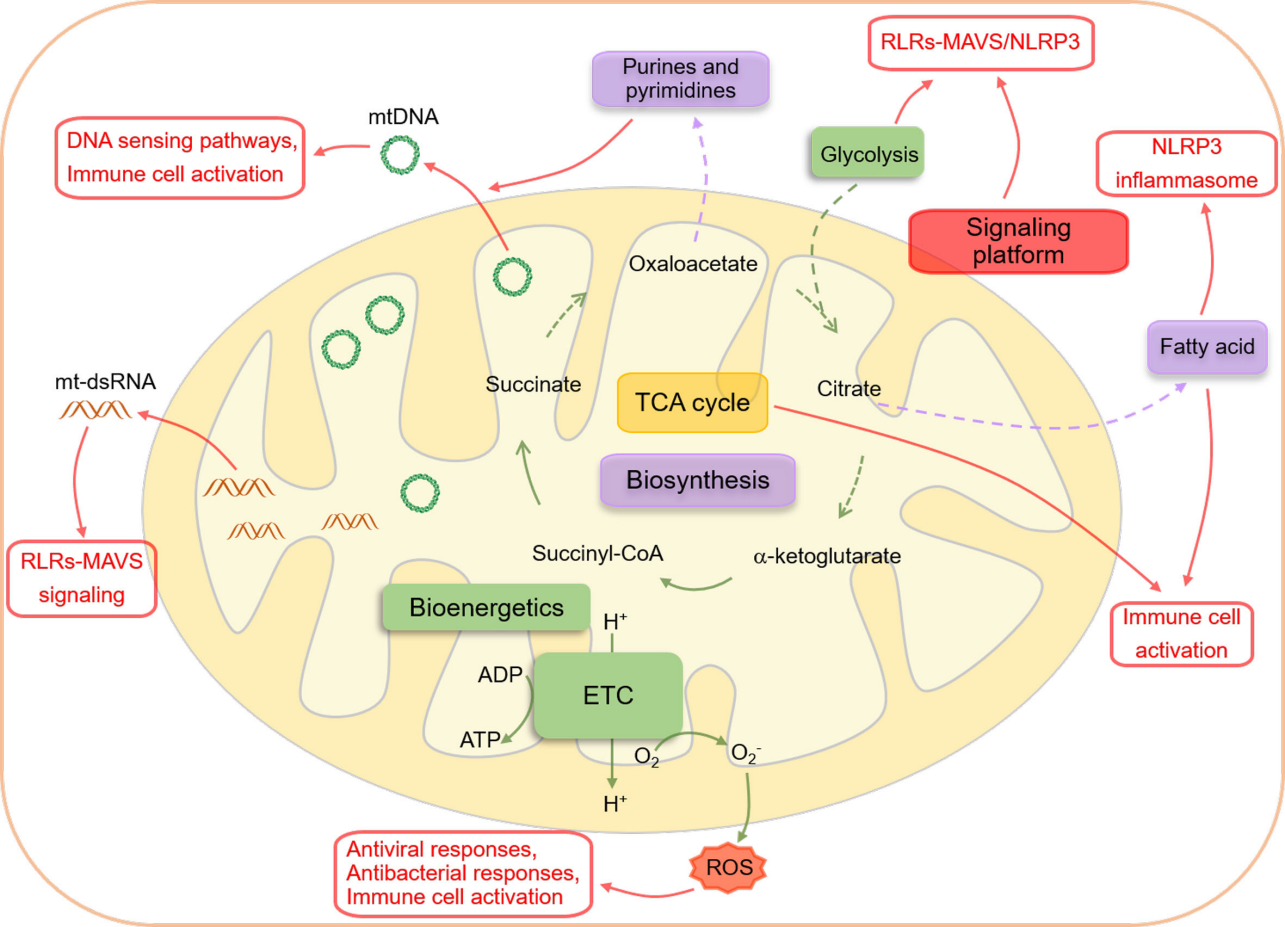
An overview of interactions between mitochondria and innate immune responses. Mitochondria are essential metabolic organelles that play an important role in maintaining cellular energy homeostasis through efficiently coupling the TCA cycle to the ETC. The TCA cycle, initiated by acetyl-CoA generated from glycolysis-derived pyruvate dehydrogenation or fatty acid oxidation, produces NADH and FADH2, which supply electrons to the ETC for ATP production (bioenergetics). The intermediates of the TCA cycle also participate in biomacromolecule generation, including glycogen, lipids, nucleotides, and proteins, through anabolic pathways (biosynthesis). In addition to their metabolic functions, mitochondria also serve as signaling hubs that regulate various cellular biological events, particularly immune responses, through several mechanisms. Firstly, the mitochondrial outer membrane acts as a platform for the aggregation of MAVS and the formation of the NLRP3 inflammasome, facilitating the RLRs-MAVS and NLRP3 inflammasome signaling pathways. Secondly, the cytoplasmic release of mtDNA and mtRNA from dysfunctional mitochondria can be recognized by PRRs and directly trigger innate immune responses. Finally, mitochondrial metabolites from the TCA cycle and metabolic byproducts, such as ROS, can precisely modulate the activation of innate immunity.

The mitochondrion is a functionally versatile organelle that has evolved from α-proteobacterium, a prokaryotic organism (1–3). According to the endosymbiont theory, an archaeon engulfed this bacterium about 2 billion years ago, forming a symbiotic relationship to meet its nutritional needs (2, 4–6). This bacterial origin of mitochondria probably explains innate immune responses triggered by recognizing unique mitochondrial components by various receptors (7). Modern mitochondria consist of five distinct components, including an outer and inner membrane, an intermembrane space, cristae formed by the infoldings of the inner membrane, and a matrix (4). One of the critical differences between mitochondria and other cellular organelles is that mitochondria have mitochondrial DNA (mtDNA), the circular DNA that encodes 13 proteins necessary for oxidative phosphorylation complexes formation, 22 ribosomal RNAs, and 2 transfer RNAs required for mitochondrial RNA (mtRNA) translation (8, 9). Mitochondria are also highly dynamic organelles that change rapidly to meet the demands of various cellular processes (10, 11) *via* the balance of mitochondrial fusion and fission, which is crucial in regulating cellular metabolism, calcium homeostasis, reactive oxygen species (ROS) generation, and mitochondrial quality control (12). Mitochondria are considered the bioenergetic organelles and biosynthetic hubs that use glycolysis-derived pyruvate, fatty acids, and amino acids to generate adenosine triphosphate (ATP) *via* the oxidative phosphorylation process to maintain cellular homeostasis (4) and the intermediate producers for anabolic pathways (4, 13). However, a wide variety of studies have also illustrated mitochondria as signaling hubs that regulate numerous cellular biological events, including metabolism, cell fate determination, and immune responses through forming signaling platforms and releasing mitochondrial ROS (mtROS), mtDNA, and metabolites (4) (**Figure 1**).

The innate immune system, *via* a plethora of pattern recognition receptors (PRRs) such as Toll-like receptors (TLRs), nucleotide-binding oligomerization domain (NOD)-like receptors (NLRs), C-type lectin receptors (CLRs), retinoic acid-inducible gene I (RIG-I)-like receptors (RLRs), and DNA sensors, is the first defense line against numerous microorganism invasions and sterile damages, such as those caused by necrotic cells-derived damage-associated molecular patterns (DAMPs) and cytokines (14–16). PRRs recognize distinct pathogen-associated molecular patterns (PAMPs) from pathogenic agents and DAMPs from damaged cells and tissues (17–19). Innate immune responses initiated by PRRs lead to significant production and processing of type I interferons (IFNs), cytokines, and proinflammatory chemokines, which modulate specific adaptive immune responses to eliminate pathogenic agents, repair damaged tissues, and maintain homeostasis (16–18). Mitochondrial functional components such as mtROS, mtDNA, cardiolipin, and the mitochondrial outer membrane (MOM) can directly activate or modulate innate immune responses (7). Meanwhile, dysfunctional mitochondria are involved in multiple inflammatory diseases (20), such as rheumatoid arthritis (RA) (21, 22), systemic lupus erythematosus (SLE) (23, 24), Sjögren’s syndrome (25), neurodegenerative diseases (26–28), fibrotic diseases (29), and aging (30, 31). This review will focus on the crucial roles of mitochondria in various innate immune responses, including cytosolic nucleic acid sensing pathways (RLR-mitochondrial antiviral signaling protein (MAVS), and cGAS-STING), inflammasomes, TLRs signaling pathways, and immune cell activation (**Figure 1**).

## 2 Mitochondria act as platforms for signaling complex assembly

### 2.1 RLRs-MAVS signaling

**Figure 2:**
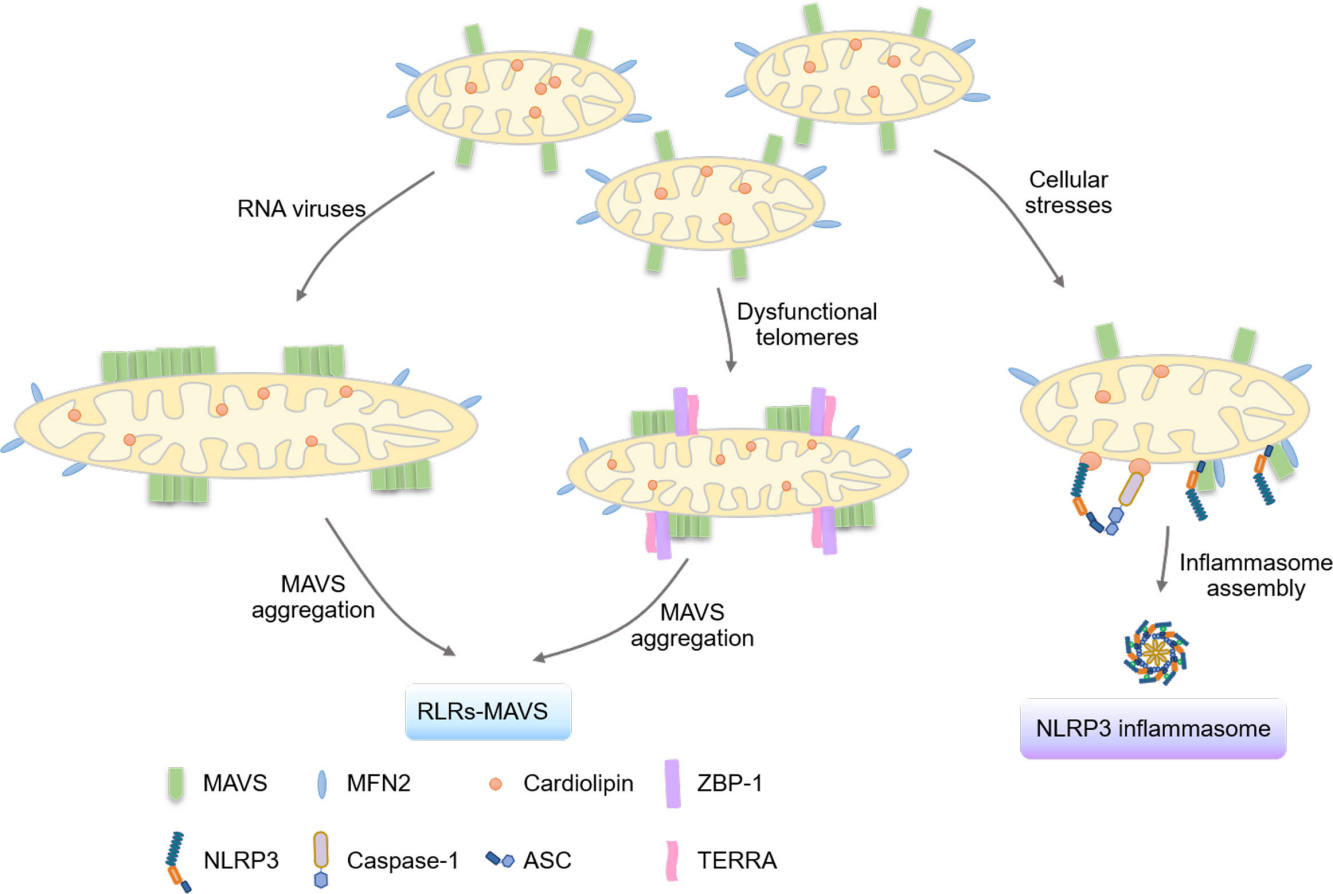
Mitochondria serve as platforms for signalosome formation. The mitochondrial outer membrane is critical in activating the innate immune system by serving as the bridging platform for the aggregation of the MAVS protein and the formation of the NLRP3 inflammasome. Upon RNA virus infection, the protein TBK1 phosphorylates and inactivates the mitochondrial fission protein DRP1, critical for the fusion of mitochondria and the aggregation of MAVS for activation. Moreover, the mitochondrial outer membrane serves as a platform for the localization of TERRA-ZBP1 complexes formed under dysfunctional telomeres, leading to the activation of the MAVS pathway. Additionally, mitochondrial proteins such as cardiolipin, MAVS, and MFN2 have been shown to regulate the assembly of the NLRP3 inflammasome by providing binding sites for NLRP3.

Mitochondria are considered as critical signal platforms to facilitate the RLRs-MAVS signaling cascade by mediating the prion-like aggregation of MAVS (32) (**Figure 2**). Apart from nucleic acids-sensing TLRs located in endosomes (33), viral RNA can be recognized by RNA sensors in the cytoplasm (34). RIG-I (35) and melanoma differentiation-associated gene 5 (MDA5) (36), which belong to the DExD/H box RNA helicase family, are identified to detect cytosolic viral dsRNA. Upon viral dsRNA association, the conserved caspase activation and recruitment domains (CARDs) of these RNA sensors (37) are exposed and bind to the CARD domains of MAVS (38–41) located in the mitochondrial outer membrane, which drives the prion-like aggregation and activation of MAVS (32, 42). This aggregation recruits kinases TANK-binding kinase 1 (TBK1)/inhibitor of nuclear factor kappa-B kinase ϵ (IKKϵ) to activate the downstream transcriptional factors, interferon regulatory factor 3/7 (IRF3/7), and nuclear factor-κB (NF-κB). mRNAs of type I/III IFNs, interferon-stimulated genes (ISGs), and proinflammatory cytokines (43) are then transcribed and translated to restrict microbial infection, modulate adaptive immunity, and initialize tissue regeneration (44, 45). Intriguingly, the peroxisome (46, 47) and mitochondrial-associated endoplasmic reticulum membranes (MAM) (48) localization of MAVS and MAVS signalosomes have also been suggested, which induce or modulate the expression of ISGs and type III IFNs. Aberrant activation of RLRs-MAVS signaling is known to cause various autoimmune and autoinflammatory disorders (49).

Proteins that regulate mitochondrial dynamics (12), including the dynamin-related family of large GTPases mitofusin 1 (MFN1), mitofusin 2 (MFN2), optic atrophy 1 (OPA1), and dynamin-related protein 1 (DRP1), play critical roles in MAVS function. MFN1 facilitates the redistribution of MAVS on mitochondria to positively regulate RLR-MAVS signaling (50), while conversely, MFN2 represses MAVS aggregation (51). Additionally, both mitochondrial membrane potential (ΔΨ(m)) and membrane proteins can function as negative regulators of MAVS (7), such as NLR family member X1 (NLRX1), globular head domain of complement component C1q receptor (gC1qR), and Polo-like kinase 1 (PLK1). Activation of RLRs-MAVS signaling, in return, appears to contribute to the elongation of the mitochondrial network *via* interaction between MAVS and MFN1 (50, 52). Notably, disrupting the mitochondrial fragmentation function of DRP1 by TBK1-mediated S412 phosphorylation forms hyper-fused mitochondrial networks, which are required to effectively assemble large MAVS aggregates during innate RNA sensing (45). In this scenario, phosphorylation of DRP1 by TBK1 directly blocks the high-order oligomerization and mitochondrial division function of DRP1 (45). The TBK1-DRP1 axis also participates in nutrient-triggered mitochondrial dynamics and cell fate determination, suggesting that innate immunity also contributes to governing the morphology and physiology of mitochondria (45).

### 2.2 NOD-like receptor pyrin domain-containing 3 inflammasomes

Inflammasomes are multi-subunit complexes activated under stress conditions to regulate inflammatory responses and induce pyroptotic cell death (53, 54). They consist of a receptor (NLR family and PYHIN protein family members), the adaptor protein ASC (apoptosis-associated speck-like protein containing a CARD), and the inflammatory cysteine protease pro-caspase 1 (55). Mitochondria act as scaffolds for NLRP3 inflammasome assembly. NLRP3 translocates from the endoplasmic reticulum (ER) to mitochondria and the MAM to form the NLRP3 inflammasome, which leads to the maturation of caspase-1-dependent proinflammatory cytokines IL-1β and IL-18 (56, 57). Cardiolipin, MAVS, and MFN2 have also been shown to regulate NLRP3 inflammasome assembly. For instance, upon mitochondrial stresses, cardiolipin, the mitochondrial inner membrane-associated phospholipid, is exposed on the mitochondrial surface to serve as the independent binding site for NLRP3 and full-length caspase-1 to assemble and activate the inflammasome (58, 59). Alternatively, MAVS forms a complex with MFN2 (60) to recruit NLRP3 to the mitochondria for NLRP3 inflammasome assembly during RNA viral infection (61–63).

### 2.3 ZBP1-mediated signaling

A recent study indicated the importance of mitochondrial signal platforms in telomere-mediated tumor suppression and aging. Z-conformation nucleic acid binding protein 1 (ZBP1) (64), an IFN-stimulated gene, functions as a cytosolic Z-nucleic acid sensor to regulate type I IFN signaling, inflammation, cell death, and tissue homeostasis (65). The study showed that mitochondria provide a scaffold for ZBP1-telomeric-repeat-containing RNAs (TERRA) complexes to activate MAVS-dependent interferon response during a replicative crisis (66).

These findings suggest that mitochondrial architecture, rather than a single mitochondria-related protein or product, is essential in maintaining various innate immune responses, which provide a broader perspective for studying the relationship between mitochondria, immunity, and diseases.

## 3 Regulation of innate immune responses by mitophagy

Maintaining mitochondrial health is crucial for properly functioning the immune system (67, 68). Two primary pathways for dealing with damaged mitochondria are proposed, including mitochondrial quality control mechanisms to immediately process defective or misfolded/mislocalized mitochondrial proteins and mitophagy that delivers irreversibly damaged mitochondria to the lysosome for degradation (31, 69). Mitophagy is mainly controlled by the ubiquitin (Ub)-dependent [PTEN-induced kinase 1 (PINK1)/Parkin RBR E3 ubiquitin-protein ligase (Parkin)] (70) or Ub-independent (specific LIR-containing receptor-dependent) pathways, such as those mediated by BNIP3 (BCL2 interacting protein 3), BNIP3L (BNIP3-like, also called NIX), FUNDC1 (FUN14 domain containing 1), PHB2 (prohibitin 2), BCL2L13 (BCL2-like protein 13), and FKBP8 (FK506-binding protein prolyl isomerase 8) (67, 71).

### 3.1 Mitophagic regulation of IFN signaling

Mitophagy plays a crucial role in regulating type I IFN signaling activation. Deficient mitophagy caused by autophagy related 5 (ATG5) ablation increases mtROS production and elevates levels of MAVS, promoting the activation of the type I IFN pathway (72). Sequestosome 1 (SQSTM1/p62)-dependent mitophagy (73) and mitophagy induced by viral proteins (71, 74) also regulate the type I IFN response. By contrast, NIX-dependent mitophagy acts as an intrinsic negative regulator of the RLRs-MAVS axis by preventing spontaneous aggregation of endogenous MAVS in the absence of viral infection (75). Besides, the disruption of mitophagy caused by PINK1 and Parkin mutations contributes to the activation of the cGAS-STING signaling pathway *via* cytosolic accumulation of mtDNA (76), which will be elaborated on later.

### 3.2 Mitophagic regulation of inflammasomes

It has been found that mitophagy helps to repress NLRP3 inflammasome activation by reducing mtROS production and mtDNA release *via* clearing damaged mitochondria (57, 67), while Parkin plays a crucial role in mtROS-NLRP3-mediated inflammatory response by regulating mitophagy activation (77). Defective mitophagy and mtROS accumulation induced by receptor-interacting protein kinase 2 (RIPK2) deletion lead to increased morbidity and mortality by accelerating IL-18 secretion and inflammatory activation during influenza A virus (IAV) infection (78). NF-κB signaling is also vital in anti-inflammatory by inducing the expression of SQSTM1/p62 to promote autophagic clearance of damaged mitochondria in lipopolysaccharide (LPS)-treated macrophages (79). Besides Ub-dependent mitophagy, FUNDC1-mediated Ub-independent mitophagy can control the secretion of inflammasome-related IL-1β (80). Additionally, mitophagy regulates the activation of AIM2 (absent in melanoma 2) (81) and NLRC4 (NLR-family CARD domain-containing protein 4) (82) inflammasomes, revealing a widespread role of mitophagy in controlling inflammasome-mediated innate immune responses.

## 4 Regulation of innate immune responses by mitochondrial apoptosis

Mitochondria are required to regulate cell apoptosis through the intrinsic pathway, which facilitates many biological processes, including the regulation of inflammatory responses (83, 84). During mitochondrial apoptosis induced by various cellular stresses, the pro-apoptotic effectors BCL2-associated X protein (BAX) and BCL2 homologous antagonist/killer protein (BAK) are activated by BH3-only proteins to assemble the mitochondrial outer membrane permeabilization (MOMP) (84, 85). MOMP allows the release of mitochondrial soluble proteins to activate apoptotic caspases, including cytochrome *c* which binds to apoptotic peptidase activating factor 1 (APAF1) to form the apoptosome (86), second mitochondrial-derived activator of caspases (SMAC) and high-temperature requirement protein A2 (HtrA2/OMI) which induce the degradation of the caspase inhibitor XIAP (X-linked inhibitor of apoptosis protein) (84).

Mitochondrial apoptosis impacts the type I IFN pathway and inflammation *via* MOMP in several ways (84). Under conditions of caspase deficiency, stimuli induce mitochondrial apoptosis to promote NF-κB signaling through the upregulation of NF-κB-inducing kinase (NIK) resulting from MOMP formation and inhibitor of apoptosis proteins (IAPs) degradation (87). This phenotype has also been observed with SMAC-mimetic compounds treatment (88, 89), but an exception exists. Due to the redundancy of other mitochondrial IAP binding proteins, deletion of SMAC and OMI fails to prevent MOMP-induced IAPs degradation (90, 91). Therefore, further research is needed to explain the mechanism of IAPs depletion by MOMP. Interestingly, MOMP-related IAPs degradation in macrophages can activate caspase 8 to promote the maturation of IL-1β and the activation of NLRP3 inflammasome (92, 93). In addition, the activation of apoptotic caspases-induced potassium efflux also contributes to NLRP3 inflammasome formation (94). Furthermore, mitochondrial nucleic acids, such as mtDNA and mtRNA, released through MOMP, mitochondrial permeability transition pore (MPTP), or voltage-dependent anion-selective channels (VDAC), can robustly activate cytosolic nucleic acids sensing pathways, which will be discussed in the next section. However, the exact role and nature of the mitochondrial inner membrane during these molecular events are still poorly understood.

It is worth noting that in most conditions, mitochondrial apoptosis is non-inflammatory. Apoptotic caspases have been found to inhibit inflammation by directly cleaving inflammatory components such as MAVS, cGAS, and IRF3 (95). Additionally, they can inhibit protein translation and canonical protein secretory processes (84, 96) and induce rapid cell death to remove damaged cells (97). However, other apoptotic caspase-independent mechanisms also contribute to MOMP-dependent anti-inflammatory responses. For example, MOMP induces the release of PNPase-polynucleotide phosphorylase (PNPT1), which degrades mtRNA to block the RLRs-MAVS pathway (98). Mitophagy is another effective approach to maintaining non-inflammatory apoptosis (99). Despite the identification of mitochondrial apoptosis in regulating innate immunity through MOMP, more in-depth studies are still needed to understand the specific pathways and pores or channels associated with the release of mitochondrial-related DAMPs.

## 5 Innate immune responses triggered by mtDNA

**Figure 3:**
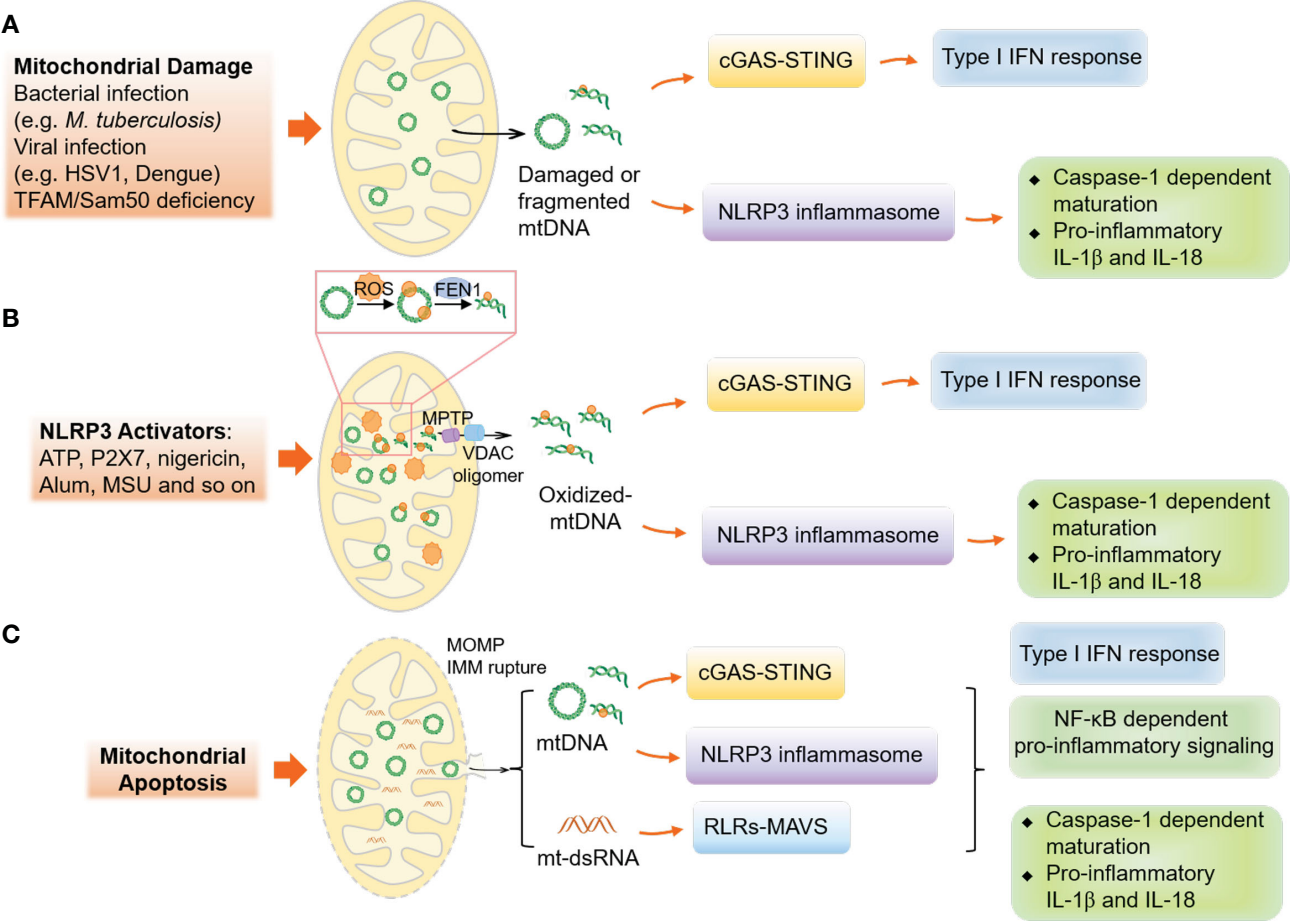
Mechanisms of mitochondrial DNA release and mtDNA-associated innate immune responses. **(A)** The disruption or stress of mitochondria, caused by infections like bacterial or viral, can lead to the release of damaged or fragmented mtDNA into the cytoplasm, activating the cGAS-STING and NLRP3 inflammasome responses. **(B)** In non-apoptotic cells, the activation of NLRP3 results in oxidative stress on mitochondria, causing the modification and cleavage of mtDNA by the endonuclease FEN1. The MPTP and VDAC oligomers in the mitochondrial outer membrane facilitate the release of fragmented mtDNA. **(C)** In apoptotic cells, the formation of BAX and BAK oligomers leads to MOMP, causing the release of mtDNA and mt-dsRNA and activating innate immune responses.

mtDNA is located in the mitochondrial matrix, which associates with the necessary cofactors for mtDNA transcription, replication, and repair (100–103). In 2004, Collins et al. by injecting mtDNA into the joints of mice, demonstrated that oxidized mtDNA might play a role in inflammation, which resulted in inflammation and arthritis (104). This discovery led to a new avenue of research on how mtDNA functions as a critical DAMP under stress conditions. Various innate immune receptors have been known to recognize mtDNA for initiating innate immune responses, as shown in **Figure 3**.

### 5.1 Mechanisms of mtDNA release

Although a wide variety of studies have demonstrated the importance of cytosolic mtDNA in innate immunity, the mechanisms explaining the translocation of mtDNA from the mitochondrial matrix to the cytosol are still not well understood. In 2014, two independent studies uncovered that mtDNA is released during mitochondrial apoptosis (105, 106). Follow-up studies demonstrated that apoptosis-related MOMPs allow the rupture of the mitochondrial inner membrane and mtDNA release (107–110). Additionally, under subtle stress-induced non-apoptotic conditions, only a fraction of mitochondria undergoing permeabilization (minority MOMPs) also cause the cytosolic release of mtDNA and the activation of cGAS-STING signaling, the major pathway sensing of mtDNA in the cytosol (111, 112). Furthermore, VDAC, which is used for metabolites and ions transport (113), can oligomerize to form pores in the MOM under oxidative stress conditions (114) and interact with mtDNA and form oligomers in the MOM to permit the release of short mtDNA fragments and trigger type I IFN response (115), while MPTP, formed in the mitochondrial inner membrane under various cellular stress conditions, is considered to regulate the release of small mtDNA fragments (116, 117). Notably, cytoplasmic accumulation of TAR DNA binding protein 43 (TDP-43), a disease hallmark of amyotrophic lateral sclerosis (ALS), can trigger mtDNA release *via* the MPTP and VDAC1 oligomers to upregulate the NF-κB and cGAS-STING signaling (118). Meanwhile, the endonuclease Flap endonuclease 1 (FEN1) has been found to cleave oxidized mtDNA into 600-650 bp fragments that can be released from mitochondria *via* MPTP and VDAC-dependent channels (119). Although MOMP-induced mtDNA release in apoptotic cells has been discussed in detail, the regulations of mtDNA release in living cells under moderate-level stress and the role of the mitochondrial inner membrane in mtDNA release are still not well understood. These mechanisms of mtDNA release are summarized in **Figure 3**.

### 5.2 mtDNA triggers cGAS-STING signaling

cGAS is a primary cytosolic DNA receptor that can recognize both exogenous and endogenous DNA and induce the production of 2’3’-cyclic GMP-AMP (cGAMP) (120, 121), which activates the adaptor protein STING (122–125) to elicit type I IFN and inflammatory responses (126). In addition to its role in response to viral and bacterial DNAs (127–131), the cGAS-STING pathway also elicits and controls self-DNA (nuclear DNA and mtDNA)-induced innate immune responses (132), inflammatory diseases (133, 134), antitumor immunity (135–137), neurodegenerative diseases (138), and diverse cellular functions (139) including protein synthesis (140) and glucose metabolism (141).

Various signals can disrupt mitochondrial integrity during pathogen infections. For instance, IAV triggers the release of mtDNA through the proton-selective ion channel (viroporin) activity of the M2 protein in a MAVS-dependent manner and evades the recognition of cGAS by interacting with NS1 proteins and mtDNA (142). Infections of herpes simplex virus 1/2 (HSV-1/HSV-2) have been found to cause stress and elimination of mtDNA, which regulates antiviral responses and ISG expression (143–146). Interestingly, infections with RNA viruses, such as Dengue virus, can activate the cGAS-STING (147–149) and TLR9 pathways (150) by causing mitochondrial stress and oxidized mtDNA release. Certain strains of *Mycobacterium tuberculosis* can also trigger cGAS-STING activation (128, 130, 151) by inducing mtDNA release (152). During pathogen infections, IL-1β secretion can upregulate antimicrobial immune responses by releasing mtDNA to activate the cGAS-STING signaling pathway (153), providing new insights into the mechanisms by which numerous cytokine-related pathways boost inflammatory responses. These results suggest that maintaining mtDNA homeostasis may be a beneficial regulator of innate antiviral immunity.

In addition to pathogen infection, the disruption of mitochondrial DNA integrity, replication, and repair can activate the cGAS-STING pathway (133). For instance, mitochondrial transcription factor A (TFAM) deficiency can cause cGAS-STING activation by damaging the nucleoid structure and accumulating cytosolic mtDNA (8, 145, 154), while depletion of sorting and assembly machinery component 50 (Sam50) (155) and endonuclease G (115) similarly induces mtDNA-related cGAS-STING activation in hepatocytes and other cells. Additionally, chemotherapeutic drugs (156), pyrimidine nucleotide carrier SLC25A33 overexpression, as well as the inhibition of *de novo* pyrimidine synthesis (157), can trigger mtDNA leaking and cGAS-STING activation. As a result, the importance of cGAS-STING in sensing mtDNA and inflammatory-related diseases has made it a highly targeted drug (158).

### 5.3 mtDNA serves as an inflammasome activator

Nakahira et al. were the first to report that the MPTP-induced cytosolic accumulation of mtDNA and mtROS strengthens NLRP3 inflammasome activation in autophagic protein-deficient macrophages (159). mtDNA oxidized by mtROS is preferred for NLRP3 recognition (79, 160–162). Activating inflammasomes and caspases also regulate mtDNA release by causing mitochondrial damage. Notably, the role of NLRP3 may not promote mtDNA release but rather stabilize it in the cytoplasm (162), while other studies indicate that NLRP3 activation amplifies the mitochondrial damage and mtDNA release (163, 164). Caspase-1 activated by inflammasomes destroys mitochondria by triggering multiple pathways to promote the production of mtROS, the dissipation of ΔΨ(m), and the permeabilization of mitochondrial membranes (163). Moreover, NLRP3 promotes caspase-2 activation and BID cleavage during infection-related ER stress to facilitate mitochondrial permeabilization (164). Further studies are therefore needed to clarify those conflicting data and better understand the relationship between mtDNA and NLRP3.

### 5.4 mtDNA and TLR9

TLR9 is a member of the TLRs family and is the first TLR discovered to sense bacterial DNA with hypomethylated CpG motifs, and this recognition activates the MAPK and NF-κB signaling pathways, leading to an inflammatory response (134, 165). Interestingly, TLR9 has also been found to recognize mtDNA released into the bloodstream during systemic inflammatory response syndrome (SIRS) and activate a p38-mediated inflammatory response (166, 167). mtDNA released from dying cells can form a complex with the antimicrobial peptide LL-37 to evade the degradation by DNase II and activate TLR9 response (168). In addition, the mtDNA-TFAM complex released from necrotic cells augments proinflammatory response by promoting the activation of receptor for advanced glycation end products (RAGE) and TLR9 (169, 170). Mitochondrial dynamics can also play a role in TLR9-induced inflammation by affecting mtDNA stability (171, 172). The absence of OPA1 in mice results in muscle atrophy and premature death due to the accumulation of damaged mitochondria and disruption of mitophagy, leading to mtDNA-related TLR9-mediated inflammation (173). Circulating mtDNA has been confirmed as an endogenous TLR9 agonist in various studies and has been implicated in several inflammatory-related diseases (9), including rheumatoid arthritis (22), atherosclerosis (168, 174), hypertension (175), acute liver injury (176) and non-alcoholic steatohepatitis (177).

### 5.5 mtDNA and neutrophil extracellular traps

mtDNA can also play a role in the extracellular space by engaging the cGAS-STING pathway and/or the TLR9 pathway on neighboring immune cells (24, 178, 179), such as in the scenario of neutrophil extracellular traps (NETs) during microbial infection and sterile inflammatory diseases (9, 180). NETs are vast extracellular decondensed-chromatin networks containing a plethora of microbial-killing proteins and DNAs (180, 181). In healthy neutrophils with oxidative damage, entire TFAM-mtDNA complexes are expelled into the extracellular space, and mtDNA can be transported into lysosomes to avoid recognition by TLR9 and maintain the immunological silence of plasmacytoid dendritic cells (pDCs) (179). Conversely, oxidized mtDNA is released in systemic lupus erythematosus (SLE) patients and activates inflammatory responses (179). The formation of oxidized mtDNA-containing NETs can be stimulated by ribonucleotide immune complexes (RNP-ICs) (178) and continuous IFN-α signaling (183), which further strengthens the cGAS-STING pathway. Additionally, lymphocytes and eosinophils can engage the type I IFN response in peripheral blood mononuclear cells by secreting mtDNA-containing webs (184, 185). These findings highlight the importance of mtDNA in both intracellular and extracellular pathways in regulating immune responses.

## 6 mtRNA in triggering RLR-MAVS signaling

The mitochondrial genome contains both heavy (H) and light (L) strands for the transcription of functional RNAs and several non-coding RNAs (186, 187). Under normal conditions, non-coding RNAs are degraded by the RNA degradosome to prevent the formation of mitochondrial double-stranded RNA (mt-dsRNA) (188, 189). However, during MOMP, mt-dsRNA can be released into the cytoplasm (110, 190), and recognized by MDA5, leading to the activation of the type I IFN response (110, 190). Interestingly, dysfunction of the mtRNA degradosome component, PNPase, can lead to the activation of the type I IFN response by causing the accumulation of mt-dsRNA (190, 191). Additionally, the protein kinase R (PKR) can also detect mt-dsRNA under stress conditions (192) (**Figure 3**).

## 7 Regulation of innate immunity by mitochondrial metabolism

The metabolites generated from glycolysis, the electron transport chain (ETC), and the tricarboxylic acid (TCA) cycle play a crucial role in regulating innate immunity, including the type I IFN response, the NLRP3 inflammasome, and immune cell activation.

### 7.1 mtROS in innate immune responses

**Figure 4:**
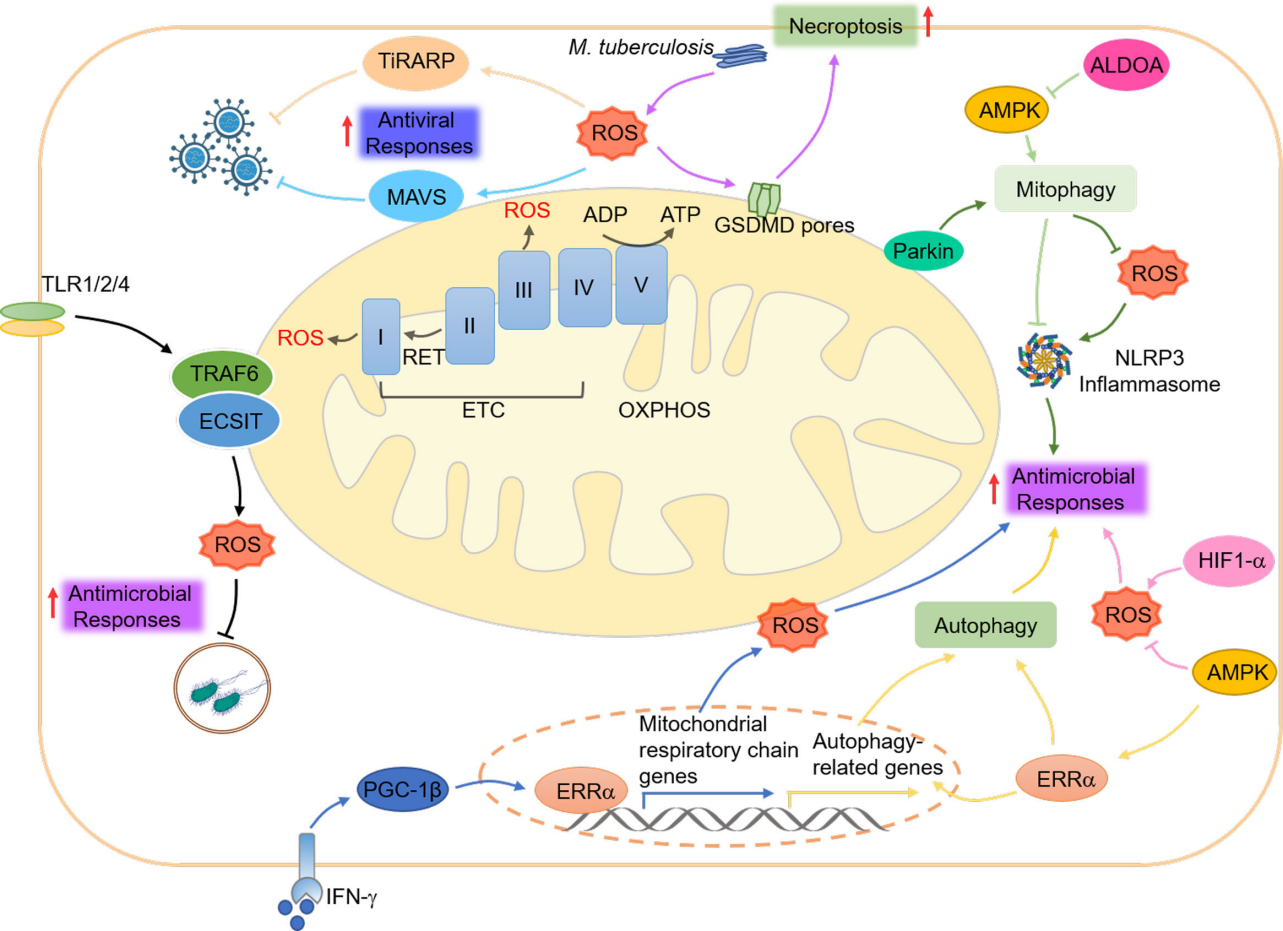
Mitochondrial ROS and innate immunity. mtROS is produced at complexes I and III of the ETC in response to hypoxia, changes in substrate availability, and abnormal mitochondrial or cellular conditions. mtROS plays a role in coordinating innate immune responses, including antiviral signaling through the RLRs-MAVS, antimicrobial responses through the NLRP3 inflammasome and TLR pathways, and necroptosis through GSDMD. The cellular metabolism regulator AMPK helps to maintain a balance of mtROS and promote antimicrobial responses by inhibiting their generation, while HIF1-α enhances their production. AMPK also regulates the activation of the NLRP3 inflammasome by suppressing mtROS and promoting autophagy, which ERRα regulates through post-translational and transcriptional mechanisms. Additionally, the key regulator of mitophagy, Parkin, controls mtROS production and the activation of the NLRP3 inflammasome. Finally, increased cytosolic mtROS have been found to drive the mitochondrial localization of GSDMD, leading to the formation of a mitochondrial GSDMD pore and the acceleration of necroptosis.

mtROS, the “so-called” byproducts of the mitochondrial respiration chain, is generated at complexes I and III of the ETC in response to hypoxia, substrate availability alteration, and abnormal mitochondrial conditions (193). mtROS has been demonstrated to play a crucial role in innate immunity (194) (**Figure 4**).

#### 7.1.1 mtROS and innate antiviral response

Mitophagy has been shown to regulate mtROS production during viral infection, promoting the RLRs-MAVS signaling (72). In this scenario, cytochrome *c* oxidase 5 b (COX5B), a subunit of the cytochrome *c* oxidase complex, serves as a negative feedback effector of the RLRs-MAVS pathway by repressing mtROS generation upon Sendai virus (SeV) infection (195). On the other hand, the IAV protein M2 positively regulates MAVS aggregation by controlling mtROS production (196). Additionally, the zinc finger protein tetrachlorodibenzo-p-dioxin (TCDD)-inducible poly (ADP-ribose) polymerase (TiPARP) serves as a PRR for the RNA of the Sindbis virus and can be redistributed by mtROS from the nucleus to the cytoplasm, protecting against viral infection in mice (197). These intriguing findings propose the intricate interplay between mtROS and innate immune signaling pathways in response to viral infections.

#### 7.1.2 mtROS and NLRP3 inflammasomes

The activation of the NLRP3 inflammasome can be triggered by numerous PAMPs and DAMPs that depend on mtROS generation. For instance, inhibiting the function of mitochondrial respiratory complexes I and III by small molecules can induce mtROS generation and NLRP3 activation (56, 57, 198). During oxidative stress, increased mtROS and Ca^2+^ are detected, which promote the formation of MPTP, and increased mitochondrial Ca^2+^ further facilitates the production of mtROS in this situation (199, 200). mtDNA released into the cytoplasm can be oxidized by mtROS, leading to NLRP3 inflammasome activation (162). It is noted that mtROS only activates the NLRP3 inflammasome but not other inflammasome subsets (56). Aldolase A (ALDOA) also plays a role in maintaining NLRP3 inflammasome activation by restricting activation of the AMP-activated protein kinase (AMPK) and mitophagy (201).

#### 7.1.3 mtROS and neutrophil activation

mtROS also significantly contributes to neutrophil activation, such as neutrophil degranulation, NET formation (178), Ca^2+^ ionophores induced NETosis (202), and cytokines production (203). *In vitro* studies have shown that inhibiting the production of mtROS by the antioxidant SkQ1 can accelerate the apoptosis of the chemotactic peptide fMLP-activated neutrophils (204). However, in synoviocytes, reducing the mitochondrial membrane potential and increasing ROS production through methotrexate (MTX) treatment can induce mitochondrial apoptosis (205).

#### 7.1.4 mtROS in antibacterial and anti-parasite activities

Evidence suggests that mtROS functions as a crucial agent in antibacterial defense (194). For example, accumulation of mtROS has been observed in TLR1, 2, and 4-activated macrophages to enhance the bactericidal activity of these cells by activating the downstream NF-κB response (206), which is achieved through TRAF6-mediated ubiquitination and enrichment of ECSIT (evolutionarily conserved signaling intermediate in Toll pathways), a protein involved in mitochondrial respiratory chain assembly (207). Additionally, mtROS is necessary to trigger p38 signaling upon TLR4 activation (194). In addition to TLRs responses, the IFN-γ signaling pathway also boosts the production of mtROS through the nuclear receptor estrogen-related receptor α (ERRα) to clear cellular Listeria monocytogenes (208). Patients with tumor necrosis factor receptor-associated periodic syndrome are more sensitive to LPS stimulation due to increased mtROS and inflammatory cytokines (209).

The metabolic balance regulators AMPK and mechanistic target of rapamycin (mTOR) contribute to antimicrobial responses by regulating mtROS production. For example, during some pathogenic bacterial infections, AMPK inhibits mtROS production, while hypoxia-inducible factor 1α (HIF-1α) upregulates its generation to maintain proper levels and promote antimicrobial responses (210, 211). HIF-1α also combines with mTOR to control antimicrobial signaling through glycolysis, with mTOR promoting mtROS accumulation in monocytes (212, 213). However, in *Trypanosoma cruzi*-infected macrophages, mTOR inhibition increases mtROS production and NLRP3 inflammasome activation to clear cytoplasmic parasites (214). Interestingly, in *Mycobacterium tuberculosis*-infected Lrrk2^G2019S^ mice, increased cytosolic mtROS can directly associate with gasdermin D (GSDMD), a member of the plasma membrane pore-forming family involved in pyroptosis, to form mitochondrial GSDMD pore, promoting mtROS release and necroptosis, leading to hyperactivation of inflammation and severe immunopathology (215). These studies illuminate the crucial role of mtROS in precisely regulating the autophagy-inflammasome axis to control innate immune activation.

### 7.2 Glucose metabolism and MAVS signaling

**Figure 5:**
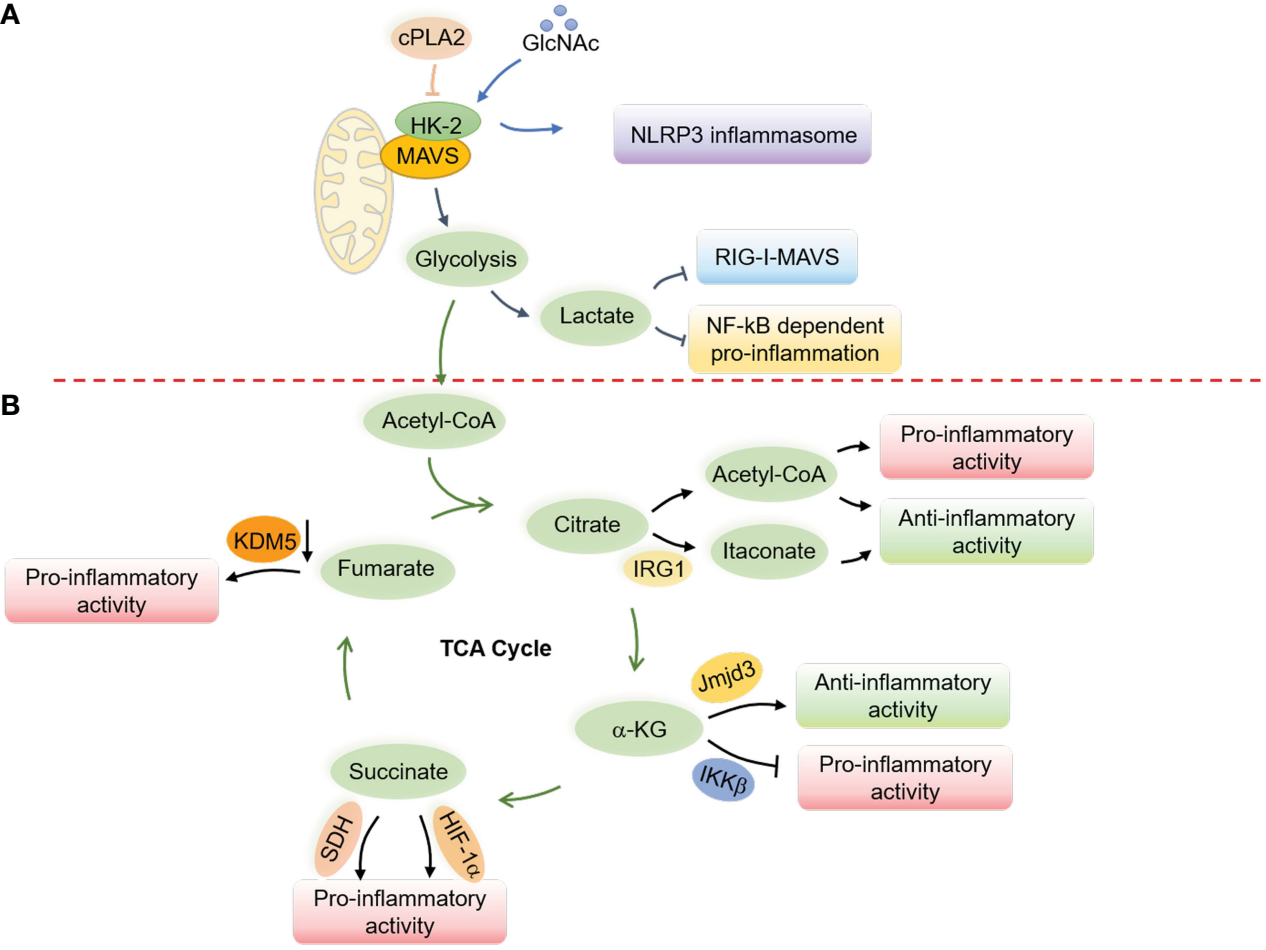
Regulation of innate immune responses and macrophage activation by mitochondrial metabolism. **(A)** Glucose metabolism regulates the RLRs-MAVS signaling and the NLRP3 inflammasome through the hexokinase 2 (HK-2) and lactate generation. In a resting state, MAVS interacts with HK-2 to maintain its kinase activity and proper glycolysis process, while lactate production interrupts the mitochondrial localization of MAVS to suppress the RLRs-MAVS signaling. cPLA2 disrupts the interaction of MAVS and HK-2, thereby boosting NF-κB-related inflammation. GlcNAc, derived from the peptidoglycan of the bacterial cell wall, can interact with HK-2 to promote its redistribution into the cytoplasm and facilitate the activation of the NLRP3 inflammasome. **(B)** Metabolites from the TCA cycle control macrophage polarization by regulating chromatin modifications, DNA methylation, and post-translational modifications of proteins. Elevated cytosolic acetyl-CoA increases histone acetylation to promote the expression of inflammatory molecules and determine macrophage polarization. Itaconate, derived from cis-aconitate, engages anti-inflammatory activity in LPS-stimulated macrophages by inhibiting mtROS production, reducing succinate dehydrogenase (SDH) activity, blocking the inhibitor of NF-κB, the NF-κB-binding protein (IκBζ), and stabilizing nuclear factor erythroid 2-related factor 2 (Nrf2). α-KG engages anti-inflammation *via* Jmjd3-dependent metabolic and epigenetic reprogramming and inhibits the proline hydroxylation of IKKβ to repress pro-inflammation. Succinate facilitates proinflammatory activity by enhancing ROS production through stabilizing HIF-1α and being oxidized by SDH. Fumarate increases proinflammatory activity by inhibiting the KDM5 histone demethylase activity, thereby promoting the gene transcription of TNFα and IL-6 cytokines.

Glucose metabolism has been shown to suppress RLR-induced interferon production through lactate, which directly binds to MAVS and disrupts its mitochondrial localization (216). MAVS directly binds to hexokinase-2 (HK-2) in its resting state to maintain its kinase activity and proper glycolysis process (216). However, research has also shown that the cytosolic phospholipase A2 (cPLA2) disrupts the interaction between MAVS and HK-2 in astrocytes, leading to increased NF-κB-related inflammation (217) (**Figure 5**). Notably, a recent study revealed a critical role of AMPK in potentiating both RLRs-MAVS and cGAS-STING signaling and antiviral responses *via* direct AMPK-mediated phosphorylation of TBK1 at S511 residue (141). These mutual interactions between glucose metabolism and innate immunity indicate an intricate and delicate network of immune responses related to mitochondria.

### 7.3 Mitochondrial metabolism and NLRP3 inflammasomes

Interplay of N-acetylglucosamine (GlcNAc) with hexokinase can lead to inflammatory responses in the host by disrupting hexokinase localization and activating NLRP3 inflammasomes (218) (**Figure 5**). Similarly, inhibiting glycolysis after the priming step through chemical treatment can activate the NLRP3 inflammasome (219). Free fatty acids (FAs) from diet or FA synthesis can activate the NLRP3 inflammasome (220–222). Therefore, activation of AMPK during fasting or caloric restriction suppresses FA-induced NLRP3 inflammasome activation by promoting autophagy and limiting ROS production (220, 223), in contrast to its role in potentiating nucleic acid signaling (141). Besides, in a state of low blood glucose, fatty acid oxidation provides energy and leads to the production of ketone bodies like β-hydroxybutyrate (BHB), which inhibit the activation of the NLRP3 inflammasome by inhibiting K^+^ efflux (224). By activating citrate synthase and inhibiting FA uptake, BHB reduces the level of mitochondrial acetylation, which represses NLRP3 inflammasome formation, mitochondrial dysfunction, and heart fibrosis (225). Butyrate, a short-chain fatty acid (SCFA), by contrast, inhibits NLRP3 activation by reducing pro-IL-1β levels (226).

### 7.4 Mitochondrial metabolism and macrophage polarization

Macrophages can be differentiated into two main distinct lineages on the type of activation signals they have received: M1 macrophages, characterized by a proinflammatory phenotype, and M2 macrophages, characterized by an anti-inflammatory and pro-fibrotic phenotype (227, 228). Studies have shown that oxygen consumption and reliance on mitochondrial metabolism differ between M1 and M2 macrophages (228–230). For example, M1 macrophages show reduced while M2 macrophages exhibit increased mitochondrial metabolism. Notably, the inhibition of the ECT through reverse electron transport (RET) increases the production of mtROS, stabilizes HIF-1α, and enhances the inflammatory response, favoring M1 macrophage polarization (228). Moreover, inhibiting fatty acid oxidation promotes M1 macrophage activation and suppresses M2 macrophage phenotypes (228).

Metabolites from the TCA cycle also play a vital role in controlling macrophage polarization by regulating macrophage chromatin modifications, DNA methylation, and protein post-translational modifications (231) (**Figure 5**). Acetyl-CoA, the starting point of the TCA cycle (231–235), provides acetyl groups for acetylation and influences the production of proinflammatory molecules such as nitric oxide (NO), ROS, and prostaglandin E2 (PGE2) in response to stimuli such as LPS (236). Acetyl-CoA in IL-4-related M2 macrophages, however, increases histone acetylation and M2 macrophage-related gene expression (237). The immune-responsive gene 1 protein (IRG1) produces itaconate (238, 239), which has been found to accumulate in response to *Mycobacterium tuberculosis* infection (182) and LPS stimulation (240) and exert anti-inflammatory effects (241). On the other hand, α-ketoglutarate (α-KG) promotes M2 polarization in LPS-stimulated M1 macrophages through epigenetic reprogramming and suppression of the NF-κB signaling pathway (242). Succinate similarly contributes to macrophage activation by stabilizing HIF-1α and increasing the transcription of IL-1β (243, 244), while fumarate accumulates in LPS-activated macrophages (243, 245) and modulates pro-inflammation by inhibiting lysine-specific histone demethylase 5 (KDM5) enzyme (246). These repeated observations indicate an essential role of mitochondrial metabolites in the polarization and activation of macrophages.

## 8 Concluding remarks and perspective

Mitochondria are essential cellular organelles that play a critical role in maintaining the energy balance of cells by linking the TCA cycle to the ETC. This efficient energy transfer from the TCA cycle to the ETC allows cells to generate ATP, an energy source for various cellular processes. In addition to their metabolic functions, mitochondria serve as signaling hubs that regulate various cellular biological events, particularly innate immune responses. This complex relationship between mitochondria and innate immunity involves several processes maintaining mitochondrial homeostasis. Firstly, mitochondria act as scaffolds for signaling molecules, facilitating the activation of innate immune responses by forming signal complexes. For instance, the aggregation of the antiviral signaling molecule MAVS on the mitochondrial outer membrane acts as a platform for forming the antiviral response. Similarly, the assembly of the NLRP3 inflammasome on the mitochondrial outer membrane serves as a platform for activating the inflammatory response. These scaffold functions of mitochondria play a crucial role in regulating innate immune responses. Secondly, mitochondrial metabolism also plays a key role in regulating innate immune responses. For example, the production of ROS and metabolites from the TCA cycle, including citrate, itaconate, and succinate, regulate the secretion of inflammatory cytokines, antimicrobial responses, and immune cell activation. The precise mechanisms underlying the regulation of these metabolic products in modulating innate immune responses are complex and still require further investigation. Finally, intracellular detritus from damaged mitochondria, such as mtDNA and mtRNA, serve as DAMPs that can directly activate antiviral and inflammatory responses. This mechanism of innate immune activation directly links mitochondrial dysfunction and innate immune responses and highlights the importance of mitochondrial homeostasis in regulating innate immunity.

Mitochondrial dysfunction lies at the heart of a wide array of human diseases, encompassing neurodegenerative conditions, chronic inflammation, autoimmune disorders, and metabolic diseases. Various interactions have been identified between innate immunity and various aspects of mitochondria, including their membranes, dynamics, components, and metabolites. Despite this, our comprehension of the role of mitochondria-related immune responses in the onset and progression of diseases remains incomplete. The specific nature of the signaling inputs and mechanisms governing mtDNA release, a major contributor to inflammatory responses, is not yet fully understood. Moreover, mitochondrial dysfunction is implicated in age-related diseases, particularly those involving the uncontrolled release of mitochondrial components such as mtDNA, ATP, succinate, and mtROS during aging. What causes the close association between mitochondrial integrity and aging, or conversely, is the loss of mitochondrial integrity a key driver of the aging process? In addition, various pathogen infections can trigger minor MOMP through non-lethal stimuli. Is this phenomenon a beneficial immune warning system or a detrimental factor in developing mitochondria-associated diseases? We anticipate that integrating cutting-edge techniques like high-throughput screening, omics analyses, and tissue imaging, together with the application of diverse genome-editing technologies, will significantly advance our understanding of the complex interplay between mitochondria, inflammation, and disease.

In conclusion, the relationship between mitochondria and innate immunity is a complex and multifaceted phenomenon involving various processes that maintain mitochondrial homeostasis, including mitochondrial metabolism, mitochondrial dynamics, and quality control. Further investigation is needed to fully understand the precise mechanisms of these interactions and their potential implications for developing novel therapeutic strategies against infectious and inflammatory diseases. Aberrant innate immune responses associated with dysfunctional mitochondria are related to various pathologies, including infectious, autoimmune, neurodegenerative, and cancerous diseases, and require further study to unravel the underlying mechanisms and develop new therapeutic targets for improving human health.

## Author contributions

SC and PX designed the review; ZL helped with the discussion; SC and PX wrote the review article. All authors have read and approved the published version of the manuscript.

## References

[1] Archibald JM. Endosymbiosis and eukaryotic cell evolution. Curr Biol (2015) 25(19):R911–21. doi: 10.1016/j.cub.2015.07.055 26439354

[2] Martin WF Garg S Zimorski V. Endosymbiotic theories for eukaryote origin. Philos Trans R Soc Lond B Biol Sci (2015) 370(1678):20140330. doi: 10.1098/rstb.2014.0330 26323761 PMC4571569

[3] Fan L Wu D Goremykin V Xiao J Xu Y Garg S. Phylogenetic analyses with systematic taxon sampling show that mitochondria branch within alphaproteobacteria. Nat Ecol Evol (2020) 4(9):1213–9. doi: 10.1038/s41559-020-1239-x 32661403

[4] Chandel NS. Mitochondria. Cold Spring Harb Perspect Biol (2021) 13(3):a040543. doi: 10.1101/cshperspect.a040543 33649187 PMC7919390

[5] Roger AJ Munoz-Gomez SA Kamikawa R. The origin and diversification of mitochondria. Curr Biol (2017) 27(21):R1177–R92. doi: 10.1016/j.cub.2017.09.015 29112874

[6] Lane N Martin W. The energetics of genome complexity. Nature (2010) 467(7318):929–34. doi: 10.1038/nature09486 20962839

[7] Banoth B Cassel SL. Mitochondria in innate immune signaling. Transl Res (2018) 202:52–68. doi: 10.1016/j.trsl.2018.07.014 30165038 PMC6218307

[8] Bonawitz ND Clayton DA Shadel GS. Initiation and beyond: multiple functions of the human mitochondrial transcription machinery. Mol Cell (2006) 24(6):813–25. doi: 10.1016/j.molcel.2006.11.024 17189185

[9] Riley JS Tait SW. Mitochondrial DNA in inflammation and immunity. EMBO Rep (2020) 21(4):e49799. doi: 10.15252/embr.201949799 32202065 PMC7132203

[10] Bereiter-Hahn J. Behavior of mitochondria in the living cell. Int Rev Cytol. (1990) 122:1–63. doi: 10.1016/S0074-7696(08)61205-X 2246114

[11] Chan DC. Mitochondrial dynamics and its involvement in disease. Annu Rev Pathol (2020) 15:235–59. doi: 10.1146/annurev-pathmechdis-012419-032711 31585519

[12] Giacomello M Pyakurel A Glytsou C Scorrano L. The cell biology of mitochondrial membrane dynamics. Nat Rev Mol Cell Biol (2020) 21(4):204–24. doi: 10.1038/s41580-020-0210-7 32071438

[13] Wellen KE Thompson CB. A two-way street: reciprocal regulation of metabolism and signalling. Nat Rev Mol Cell Biol (2012) 13(4):270–6. doi: 10.1038/nrm3305 22395772

[14] Chen GY Nunez G. Sterile inflammation: sensing and reacting to damage. Nat Rev Immunol (2010) 10(12):826–37. doi: 10.1038/nri2873 PMC3114424 21088683

[15] Zindel J Kubes P. DAMPs, PAMPs, and LAMPs in immunity and sterile inflammation. Annu Rev Pathol (2020) 15:493–518. doi: 10.1146/annurev-pathmechdis-012419-032847 31675482

[16] Li D Wu M. Pattern recognition receptors in health and diseases. Signal Transduct Target Ther (2021) 6(1):291. doi: 10.1038/s41392-021-00687-0 34344870 PMC8333067

[17] Brubaker SW Bonham KS Zanoni I Kagan JC. Innate immune pattern recognition: a cell biological perspective. Annu Rev Immunol (2015) 33:257–90. doi: 10.1146/annurev-immunol-032414-112240 PMC5146691 25581309

[18] Gong T Liu L Jiang W Zhou R. DAMP-sensing receptors in sterile inflammation and inflammatory diseases. Nat Rev Immunol (2020) 20(2):95–112. doi: 10.1038/s41577-019-0215-7 31558839

[19] Janeway CA Jr. Medzhitov R. Innate immune recognition. Annu Rev Immunol (2002) 20:197–216. doi: 10.1146/annurev.immunol.20.083001.084359 11861602

[20] Faas MM de Vos P. Mitochondrial function in immune cells in health and disease. Biochim Biophys Acta Mol Basis Dis (2020) 1866(10):165845. doi: 10.1016/j.bbadis.2020.165845 32473386

[21] Duvvuri B Lood C. Cell-free DNA as a biomarker in autoimmune rheumatic diseases. Front Immunol (2019) 10:502. doi: 10.3389/fimmu.2019.00502 30941136 PMC6433826

[22] Hajizadeh S DeGroot J TeKoppele JM Tarkowski A Collins LV. Extracellular mitochondrial DNA and oxidatively damaged DNA in synovial fluid of patients with rheumatoid arthritis. Arthritis Res Ther (2003) 5(5):R234–40. doi: 10.1186/ar787 PMC193725 12932286

[23] Caielli S Cardenas J de Jesus AA Baisch J Walters L Blanck JP. Erythroid mitochondrial retention triggers myeloid-dependent type I interferon in human SLE. Cell (2021) 184(17):4464–79.e19. doi: 10.1016/j.cell.2021.07.021 34384544 PMC8380737

[24] Wang H Li T Chen S Gu Y Ye S. Neutrophil extracellular trap mitochondrial DNA and its autoantibody in systemic lupus erythematosus and a proof-of-Concept trial of metformin. Arthritis Rheumatol (2015) 67(12):3190–200. doi: 10.1002/art.39296 26245802

[25] Barrera MJ Aguilera S Castro I Carvajal P Jara D Molina C. Dysfunctional mitochondria as critical players in the inflammation of autoimmune diseases: potential role in sjogren's syndrome. Autoimmun Rev (2021) 20(8):102867. doi: 10.1016/j.autrev.2021.102867 34118452

[26] Gambardella S Limanaqi F Ferese R Biagioni F Campopiano R Centonze D. Ccf-mtDNA as a potential link between the brain and immune system in neuro-immunological disorders. Front Immunol (2019) 10:1064. doi: 10.3389/fimmu.2019.01064 31143191 PMC6520662

[27] Lowes H Pyle A Santibanez-Koref M Hudson G. Circulating cell-free mitochondrial DNA levels in parkinson's disease are influenced by treatment. Mol Neurodegener. (2020) 15(1):10. doi: 10.1186/s13024-020-00362-y 32070373 PMC7029508

[28] Lin MM Liu N Qin ZH Wang Y. Mitochondrial-derived damage-associated molecular patterns amplify neuroinflammation in neurodegenerative diseases. Acta Pharmacol Sin (2022) 43(10):2439–47. doi: 10.1038/s41401-022-00879-6 PMC9525705 35233090

[29] Li X Zhang W Cao Q Wang Z Zhao M Xu L. Mitochondrial dysfunction in fibrotic diseases. Cell Death Discovery (2020) 6:80. doi: 10.1038/s41420-020-00316-9 32963808 PMC7474731

[30] Green DR Galluzzi L Kroemer G. Mitochondria and the autophagy-inflammation-cell death axis in organismal aging. Science (2011) 333(6046):1109–12. doi: 10.1126/science.1201940 PMC3405151 21868666

[31] Amorim JA Coppotelli G Rolo AP Palmeira CM Ross JM Sinclair DA. Mitochondrial and metabolic dysfunction in ageing and age-related diseases. Nat Rev Endocrinol (2022) 18(4):243–58. doi: 10.1038/s41574-021-00626-7 PMC9059418 35145250

[32] Hou F Sun L Zheng H Skaug B Jiang QX Chen ZJ. MAVS forms functional prion-like aggregates to activate and propagate antiviral innate immune response. Cell (2011) 146(3):448–61. doi: 10.1016/j.cell.2011.06.041 PMC3179916 21782231

[33] Lind NA Rael VE Pestal K Liu B Barton GM. Regulation of the nucleic acid-sensing toll-like receptors. Nat Rev Immunol (2022) 22(4):224–35. doi: 10.1038/s41577-021-00577-0 PMC8283745 34272507

[34] Loo YM Gale M Jr. Immune signaling by RIG-i-like receptors. Immunity (2011) 34(5):680–92. doi: 10.1016/j.immuni.2011.05.003 PMC3177755 21616437

[35] Yoneyama M Kikuchi M Natsukawa T Shinobu N Imaizumi T Miyagishi M. The RNA helicase RIG-I has an essential function in double-stranded RNA-induced innate antiviral responses. Nat Immunol (2004) 5(7):730–7. doi: 10.1038/ni1087 15208624

[36] Kang DC Gopalkrishnan RV Lin L Randolph A Valerie K Pestka S. Expression analysis and genomic characterization of human melanoma differentiation associated gene-5, mda-5: a novel type I interferon-responsive apoptosis-inducing gene. Oncogene (2004) 23(9):1789–800. doi: 10.1038/sj.onc.1207300 14676839

[37] Rehwinkel J Gack MU. RIG-i-like receptors: their regulation and roles in RNA sensing. Nat Rev Immunol (2020) 20(9):537–51. doi: 10.1038/s41577-020-0288-3 PMC7094958 32203325

[38] Seth RB Sun L Ea CK Chen ZJ. Identification and characterization of MAVS, a mitochondrial antiviral signaling protein that activates NF-kappaB and IRF 3. Cell (2005) 122(5):669–82. doi: 10.1016/j.cell.2005.08.012 16125763

[39] Meylan E Curran J Hofmann K Moradpour D Binder M Bartenschlager R. Cardif is an adaptor protein in the RIG-I antiviral pathway and is targeted by hepatitis c virus. Nature (2005) 437(7062):1167–72. doi: 10.1038/nature04193 16177806

[40] Xu LG Wang YY Han KJ Li LY Zhai Z Shu HB. VISA is an adapter protein required for virus-triggered IFN-beta signaling. Mol Cell (2005) 19(6):727–40. doi: 10.1016/j.molcel.2005.08.014 16153868

[41] Kawai T Takahashi K Sato S Coban C Kumar H Kato H. IPS-1, an adaptor triggering RIG-i- and Mda5-mediated type I interferon induction. Nat Immunol (2005) 6(10):981–8. doi: 10.1038/ni1243 16127453

[42] Peisley A Wu B Xu H Chen ZJ Hur S. Structural basis for ubiquitin-mediated antiviral signal activation by RIG-I. Nature (2014) 509(7498):110–4. doi: 10.1038/nature13140 PMC6136653 24590070

[43] Wu J Chen ZJ. Innate immune sensing and signaling of cytosolic nucleic acids. Annu Rev Immunol (2014) 32:461–88. doi: 10.1146/annurev-immunol-032713-120156 24655297

[44] Chan YK Gack MU. RIG-i-like receptor regulation in virus infection and immunity. Curr Opin Virol (2015) 12:7–14. doi: 10.1016/j.coviro.2015.01.004 25644461 PMC5076476

[45] Chen S Liu S Wang J Wu Q Wang A Guan H. TBK1-mediated DRP1 targeting confers nucleic acid sensing to reprogram mitochondrial dynamics and physiology. Mol Cell (2020) 80(5):810–27.e7. doi: 10.1016/j.molcel.2020.10.018 33171123

[46] Odendall C Dixit E Stavru F Bierne H Franz KM Durbin AF. Diverse intracellular pathogens activate type III interferon expression from peroxisomes. Nat Immunol (2014) 15(8):717–26. doi: 10.1038/ni.2915 PMC4106986 24952503

[47] Dixit E Boulant S Zhang Y Lee AS Odendall C Shum B. Peroxisomes are signaling platforms for antiviral innate immunity. Cell (2010) 141(4):668–81. doi: 10.1016/j.cell.2010.04.018 PMC3670185 20451243

[48] Horner SM Liu HM Park HS Briley J Gale M Jr. Mitochondrial-associated endoplasmic reticulum membranes (MAM) form innate immune synapses and are targeted by hepatitis c virus. Proc Natl Acad Sci U S A (2011) 108(35):14590–5. doi: 10.1073/pnas.1110133108 PMC3167523 21844353

[49] Onomoto K Onoguchi K Yoneyama M. Regulation of RIG-i-like receptor-mediated signaling: interaction between host and viral factors. Cell Mol Immunol (2021) 18(3):539–55. doi: 10.1038/s41423-020-00602-7 PMC7812568 33462384

[50] Onoguchi K Onomoto K Takamatsu S Jogi M Takemura A Morimoto S. Virus-infection or 5'ppp-RNA activates antiviral signal through redistribution of IPS-1 mediated by MFN1. PloS Pathog (2010) 6(7):e1001012. doi: 10.1371/journal.ppat.1001012 20661427 PMC2908619

[51] Y K O H T M I N Y Y S T. Mitofusin 2 inhibits mitochondrial antiviral signaling. Sci Signaling (2009) 2(84):ra47. doi: 10.1126/scisignal.2000287 19690333

[52] Castanier C Garcin D Vazquez A Arnoult D. Mitochondrial dynamics regulate the RIG-i-like receptor antiviral pathway. EMBO Rep (2010) 11(2):133–8. doi: 10.1038/embor.2009.258 PMC2828750 20019757

[53] Man SM Kanneganti TD. Converging roles of caspases in inflammasome activation, cell death and innate immunity. Nat Rev Immunol (2016) 16(1):7–21. doi: 10.1038/nri.2015.7 26655628 PMC4915362

[54] Rathinam VA Fitzgerald KA. Inflammasome complexes: emerging mechanisms and effector functions. Cell (2016) 165(4):792–800. doi: 10.1016/j.cell.2016.03.046 27153493 PMC5503689

[55] Zheng D Liwinski T Elinav E. Inflammasome activation and regulation: toward a better understanding of complex mechanisms. Cell Discovery (2020) 6:36. doi: 10.1038/s41421-020-0167-x 32550001 PMC7280307

[56] Zhou R Yazdi AS Menu P Tschopp J. A role for mitochondria in NLRP3 inflammasome activation. Nature (2011) 469(7329):221–5. doi: 10.1038/nature09663 21124315

[57] Swanson KV Deng M Ting JP. The NLRP3 inflammasome: molecular activation and regulation to therapeutics. Nat Rev Immunol (2019) 19(8):477–89. doi: 10.1038/s41577-019-0165-0 PMC7807242 31036962

[58] Elliott EI Miller AN Banoth B Iyer SS Stotland A Weiss JP. Cutting edge: mitochondrial assembly of the NLRP3 inflammasome complex is initiated at priming. J Immunol (2018) 200(9):3047–52. doi: 10.4049/jimmunol.1701723 PMC5916517 29602772

[59] Iyer SS He Q Janczy JR Elliott EI Zhong Z Olivier AK. Mitochondrial cardiolipin is required for Nlrp3 inflammasome activation. Immunity (2013) 39(2):311–23. doi: 10.1016/j.immuni.2013.08.001 PMC3779285 23954133

[60] Ichinohe T Yamazaki T Koshiba T Yanagi Y. Mitochondrial protein mitofusin 2 is required for NLRP3 inflammasome activation after RNA virus infection. Proc Natl Acad Sci U S A. (2013) 110(44):17963–8. doi: 10.1073/pnas.1312571110 PMC3816452 24127597

[61] Subramanian N Natarajan K Clatworthy MR Wang Z Germain RN. The adaptor MAVS promotes NLRP3 mitochondrial localization and inflammasome activation. Cell (2013) 153(2):348–61. doi: 10.1016/j.cell.2013.02.054 PMC3632354 23582325

[62] Franchi L Eigenbrod T Munoz-Planillo R Ozkurede U Kim YG Arindam C. Cytosolic double-stranded RNA activates the NLRP3 inflammasome *via* MAVS-induced membrane permeabilization and k+ efflux. J Immunol (2014) 193(8):4214–22. doi: 10.4049/jimmunol.1400582 PMC4185247 25225670

[63] Park S Juliana C Hong S Datta P Hwang I Fernandes-Alnemri T. The mitochondrial antiviral protein MAVS associates with NLRP3 and regulates its inflammasome activity. J Immunol (2013) 191(8):4358–66. doi: 10.4049/jimmunol.1301170 PMC3848201 24048902

[64] Fu Y Comella N Tognazzi K Brown LF Dvorak HF Kocher O. Cloning of DLM-1, a novel gene that is upregulated in activated macrophages, using RNA differential display. Gene (1999) 240(1):157–63. doi: 10.1016/S0378-1119(99)00419-9 10564822

[65] Kuriakose T Kanneganti TD. ZBP1: innate sensor regulating cell death and inflammation. Trends Immunol (2018) 39(2):123–34. doi: 10.1016/j.it.2017.11.002 PMC5863909 29236673

[66] Nassour J Aguiar LG Correia A Schmidt TT Mainz L Przetocka S. Telomere-to-mitochondria signalling by ZBP1 mediates replicative crisis. Nature (2023) 614(7949):767–73. doi: 10.1038/s41586-023-05710-8 PMC9946831 36755096

[67] Xu Y Shen J Ran Z. Emerging views of mitophagy in immunity and autoimmune diseases. Autophagy (2020) 16(1):3–17. doi: 10.1080/15548627.2019.1603547 30951392 PMC6984455

[68] Lazarou M. Keeping the immune system in check: a role for mitophagy. Immunol Cell Biol (2015) 93(1):3–10. doi: 10.1038/icb.2014.75 25267485

[69] Pfanner N Warscheid B Wiedemann N. Mitochondrial proteins: from biogenesis to functional networks. Nat Rev Mol Cell Biol (2019) 20(5):267–84. doi: 10.1038/s41580-018-0092-0 PMC6684368 30626975

[70] Lazarou M Sliter DA Kane LA Sarraf SA Wang C Burman JL. The ubiquitin kinase PINK1 recruits autophagy receptors to induce mitophagy. Nature (2015) 524(7565):309–14. doi: 10.1038/nature14893 PMC5018156 26266977

[71] Wang H Zheng Y Huang J Li J. Mitophagy in antiviral immunity. Front Cell Dev Biol (2021) 9:723108. doi: 10.3389/fcell.2021.723108 34540840 PMC8446632

[72] Tal MC Sasai M Lee HK Yordy B Shadel GS Iwasaki A. Absence of autophagy results in reactive oxygen species-dependent amplification of RLR signaling. Proc Natl Acad Sci U S A. (2009) 106(8):2770–5. doi: 10.1073/pnas.0807694106 PMC2650341 19196953

[73] Xia M Gonzalez P Li C Meng G Jiang A Wang H. Mitophagy enhances oncolytic measles virus replication by mitigating DDX58/RIG-i-like receptor signaling. J Virol (2014) 88(9):5152–64. doi: 10.1128/JVI.03851-13 PMC3993837 24574393

[74] Zhang L Qin Y Chen M. Viral strategies for triggering and manipulating mitophagy. Autophagy (2018) 14(10):1665–73. doi: 10.1080/15548627.2018.1466014 PMC6135629 29895192

[75] Qi N Shi Y Zhang R Zhu W Yuan B Li X. Multiple truncated isoforms of MAVS prevent its spontaneous aggregation in antiviral innate immune signalling. Nat Commun (2017) 8:15676. doi: 10.1038/ncomms15676 28607490 PMC5474743

[76] Sliter DA Martinez J Hao L Chen X Sun N Fischer TD. Parkin and PINK1 mitigate STING-induced inflammation. Nature (2018) 561(7722):258–62. doi: 10.1038/s41586-018-0448-9 PMC7362342 30135585

[77] Li J Ma C Long F Yang D Liu X Hu Y. Parkin impairs antiviral immunity by suppressing the mitochondrial reactive oxygen species-Nlrp3 axis and antiviral inflammation. iScience (2019) 16:468–84. doi: 10.1016/j.isci.2019.06.008 PMC6593176 31229895

[78] Lupfer C Thomas PG Anand PK Vogel P Milasta S Martinez J. Receptor interacting protein kinase 2-mediated mitophagy regulates inflammasome activation during virus infection. Nat Immunol (2013) 14(5):480–8. doi: 10.1038/ni.2563 PMC3631456 23525089

[79] Zhong Z Umemura A Sanchez-Lopez E Liang S Shalapour S Wong J. NF-kappaB restricts inflammasome activation *via* elimination of damaged mitochondria. Cell (2016) 164(5):896–910. doi: 10.1016/j.cell.2015.12.057 26919428 PMC4769378

[80] Li W Li Y Siraj S Jin H Fan Y Yang X. FUN14 domain-containing 1-mediated mitophagy suppresses hepatocarcinogenesis by inhibition of inflammasome activation in mice. Hepatology (2019) 69(2):604–21. doi: 10.1002/hep.30191 30053328

[81] Liu L Yang M Kang R Dai Y Yu Y Gao F. HMGB1-DNA complex-induced autophagy limits AIM2 inflammasome activation through RAGE. Biochem Biophys Res Commun (2014) 450(1):851–6. doi: 10.1016/j.bbrc.2014.06.074 PMC4107148 24971542

[82] Jabir MS Hopkins L Ritchie ND Ullah I Bayes HK Li D. Mitochondrial damage contributes to pseudomonas aeruginosa activation of the inflammasome and is downregulated by autophagy. Autophagy (2015) 11(1):166–82. doi: 10.4161/15548627.2014.981915 PMC4502769 25700738

[83] Tuzlak S Kaufmann T Villunger A. Interrogating the relevance of mitochondrial apoptosis for vertebrate development and postnatal tissue homeostasis. Genes Dev (2016) 30(19):2133–51. doi: 10.1101/gad.289298.116 PMC5088563 27798841

[84] Bock FJ Tait SWG. Mitochondria as multifaceted regulators of cell death. Nat Rev Mol Cell Biol (2020) 21(2):85–100. doi: 10.1038/s41580-019-0173-8 31636403

[85] Wei MC Zong WX Cheng EH Lindsten T Panoutsakopoulou V Ross AJ. Proapoptotic BAX and BAK: a requisite gateway to mitochondrial dysfunction and death. Science (2001) 292(5517):727–30. doi: 10.1126/science.1059108 PMC3049805 11326099

[86] Dorstyn L Akey CW Kumar S. New insights into apoptosome structure and function. Cell Death Differ (2018) 25(7):1194–208. doi: 10.1038/s41418-017-0025-z PMC6030056 29765111

[87] Giampazolias E Zunino B Dhayade S Bock F Cloix C Cao K. Mitochondrial permeabilization engages NF-kappaB-dependent anti-tumour activity under caspase deficiency. Nat Cell Biol (2017) 19(9):1116–29. doi: 10.1038/ncb3596 PMC5624512 28846096

[88] Varfolomeev E Blankenship JW Wayson SM Fedorova AV Kayagaki N Garg P. IAP antagonists induce autoubiquitination of c-IAPs, NF-kappaB activation, and TNFalpha-dependent apoptosis. Cell (2007) 131(4):669–81. doi: 10.1016/j.cell.2007.10.030 18022362

[89] Vince JE Wong WW Khan N Feltham R Chau D Ahmed AU. IAP antagonists target cIAP1 to induce TNFalpha-dependent apoptosis. Cell (2007) 131(4):682–93. doi: 10.1016/j.cell.2007.10.037 18022363

[90] Verhagen AM Kratina TK Hawkins CJ Silke J Ekert PG Vaux DL. Identification of mammalian mitochondrial proteins that interact with IAPs *via* n-terminal IAP binding motifs. Cell Death Differ (2007) 14(2):348–57. doi: 10.1038/sj.cdd.4402001 16794601

[91] Zhuang M Guan S Wang H Burlingame AL Wells JA. Substrates of IAP ubiquitin ligases identified with a designed orthogonal E3 ligase, the NEDDylator. Mol Cell (2013) 49(2):273–82. doi: 10.1016/j.molcel.2012.10.022 PMC3557559 23201124

[92] Chauhan D Bartok E Gaidt MM Bock FJ Herrmann J Seeger JM. BAX/BAK-induced apoptosis results in caspase-8-Dependent IL-1beta maturation in macrophages. Cell Rep (2018) 25(9):2354–68 e5. doi: 10.1016/j.celrep.2018.10.087 30485805

[93] Vince JE De Nardo D Gao W Vince AJ Hall C McArthur K. The mitochondrial apoptotic effectors BAX/BAK activate caspase-3 and -7 to trigger NLRP3 inflammasome and caspase-8 driven IL-1beta activation. Cell Rep (2018) 25(9):2339–53.e4. doi: 10.1016/j.celrep.2018.10.103 30485804

[94] Chen KW Demarco B Heilig R Shkarina K Boettcher A Farady CJ. Extrinsic and intrinsic apoptosis activate pannexin-1 to drive NLRP3 inflammasome assembly. EMBO J (2019) 38(10):e101638. doi: 10.15252/embj.2019101638 30902848 PMC6517827

[95] Ning X Wang Y Jing M Sha M Lv M Gao P. Apoptotic caspases suppress type I interferon production *via* the cleavage of cGAS, MAVS, and IRF3. Mol Cell (2019) 74(1):19–31.e7. doi: 10.1016/j.molcel.2019.02.013 30878284

[96] Julien O Wells JA. Caspases and their substrates. Cell Death Differ (2017) 24(8):1380–9. doi: 10.1038/cdd.2017.44 PMC5520456 28498362

[97] Arandjelovic S Ravichandran KS. Phagocytosis of apoptotic cells in homeostasis. Nat Immunol (2015) 16(9):907–17. doi: 10.1038/ni.3253 PMC4826466 26287597

[98] Liu X Fu R Pan Y Meza-Sosa KF Zhang Z Lieberman J. PNPT1 release from mitochondria during apoptosis triggers decay of Poly(A) RNAs. Cell (2018) 174(1):187–201.e12. doi: 10.1016/j.cell.2018.04.017 29779946

[99] Lindqvist LM Frank D McArthur K Dite TA Lazarou M Oakhill JS. Autophagy induced during apoptosis degrades mitochondria and inhibits type I interferon secretion. Cell Death Differ (2018) 25(4):784–96. doi: 10.1038/s41418-017-0017-z PMC5864185 29229994

[100] Cardon LR Burge C Clayton DA Karlin S. Pervasive CpG suppression in animal mitochondrial genomes. Proc Natl Acad Sci U S A. (1994) 91(9):3799–803. doi: 10.1073/pnas.91.9.3799 PMC43669 8170990

[101] BeckerYLCDuvvuriBFortinPRLoodCBoilardE. The role of mitochondria in rheumatic diseases. Nat Rev Rheumatol (2022) 18(11):621–40. doi: 10.1038/s41584-022-00834-z 36175664

[102] Anderson S Bankier AT Barrell BG de Bruijn MH Coulson AR Drouin J. Sequence and organization of the human mitochondrial genome. Nature (1981) 290(5806):457–65. doi: 10.1038/290457a0 7219534

[103] Fazzini F Schopf B Blatzer M Coassin S Hicks AA Kronenberg F. Plasmid-normalized quantification of relative mitochondrial DNA copy number. Sci Rep (2018) 8(1):15347. doi: 10.1038/s41598-018-33684-5 30337569 PMC6194030

[104] Collins LV Hajizadeh S Holme E Jonsson IM Tarkowski A. Endogenously oxidized mitochondrial DNA induces *in vivo* and *in vitro* inflammatory responses. J Leukoc Biol (2004) 75(6):995–1000. doi: 10.1189/jlb.0703328 14982943

[105] White MJ McArthur K Metcalf D Lane RM Cambier JC Herold MJ. Apoptotic caspases suppress mtDNA-induced STING-mediated type I IFN production. Cell (2014) 159(7):1549–62. doi: 10.1016/j.cell.2014.11.036 PMC4520319 25525874

[106] Rongvaux A Jackson R Harman CC Li T West AP de Zoete MR. Apoptotic caspases prevent the induction of type I interferons by mitochondrial DNA. Cell (2014) 159(7):1563–77. doi: 10.1016/j.cell.2014.11.037 PMC4272443 25525875

[107] McArthur K Whitehead LW Heddleston JM Li L Padman BS Oorschot V. BAK/BAX macropores facilitate mitochondrial herniation and mtDNA efflux during apoptosis. Science (2018) 359(6378):eaao6047. doi: 10.1126/science.aao6047 29472455

[108] Riley JS Quarato G Cloix C Lopez J O'Prey J Pearson M. Mitochondrial inner membrane permeabilisation enables mtDNA release during apoptosis. EMBO J (2018) 37(17):e99238. doi: 10.15252/embj.201899238 30049712 PMC6120664

[109] Ader NR Hoffmann PC Ganeva I Borgeaud AC Wang C Youle RJ. Molecular and topological reorganizations in mitochondrial architecture interplay during bax-mediated steps of apoptosis. Elife (2019) 8:e40712. doi: 10.7554/eLife.40712 30714902 PMC6361589

[110] Tigano M Vargas DC Tremblay-Belzile S Fu Y Sfeir A. Nuclear sensing of breaks in mitochondrial DNA enhances immune surveillance. Nature (2021) 591(7850):477–81. doi: 10.1038/s41586-021-03269-w 33627873

[111] Ichim G Lopez J Ahmed SU Muthalagu N Giampazolias E Delgado ME. Limited mitochondrial permeabilization causes DNA damage and genomic instability in the absence of cell death. Mol Cell (2015) 57(5):860–72. doi: 10.1016/j.molcel.2015.01.018 PMC4352766 25702873

[112] Brokatzky D Dorflinger B Haimovici A Weber A Kirschnek S Vier J. A non-death function of the mitochondrial apoptosis apparatus in immunity. EMBO J (2019) 38(11):e100907. doi: 10.15252/embj.2018100907 30979778 PMC6545560

[113] Mannella CA. VDAC-a primal perspective. Int J Mol Sci (2021) 22(4):1685. doi: 10.3390/ijms22041685 33567508 PMC7914813

[114] Keinan N Tyomkin D Shoshan-Barmatz V. Oligomerization of the mitochondrial protein voltage-dependent anion channel is coupled to the induction of apoptosis. Mol Cell Biol (2010) 30(24):5698–709. doi: 10.1128/MCB.00165-10 PMC3004265 20937774

[115] Kim J Gupta R Blanco LP Yang S Shteinfer-Kuzmine A Wang K. VDAC oligomers form mitochondrial pores to release mtDNA fragments and promote lupus-like disease. Science (2019) 366(6472):1531–6. doi: 10.1126/science.aav4011 PMC8325171 31857488

[116] Garcia N Chavez E. Mitochondrial DNA fragments released through the permeability transition pore correspond to specific gene size. Life Sci (2007) 81(14):1160–6. doi: 10.1016/j.lfs.2007.08.019 17870132

[117] Patrushev M Kasymov V Patrusheva V Ushakova T Gogvadze V Gaziev A. Mitochondrial permeability transition triggers the release of mtDNA fragments. Cell Mol Life Sci (2004) 61(24):3100–3. doi: 10.1007/s00018-004-4424-1 PMC11924515 15583871

[118] Yu CH Davidson S Harapas CR Hilton JB Mlodzianoski MJ Laohamonthonkul P. TDP-43 triggers mitochondrial DNA release *via* mPTP to activate cGAS/STING in ALS. Cell (2020) 183(3):636–49.e18. doi: 10.1016/j.cell.2020.09.020 33031745 PMC7599077

[119] Xian H Watari K Sanchez-Lopez E Offenberger J Onyuru J Sampath H. Oxidized DNA fragments exit mitochondria *via* mPTP- and VDAC-dependent channels to activate NLRP3 inflammasome and interferon signaling. Immunity (2022) 55(8):1370–85.e8. doi: 10.1016/j.immuni.2022.06.007 35835107 PMC9378606

[120] Wu J Sun L Chen X Du F Shi H Chen C. Cyclic GMP-AMP is an endogenous second messenger in innate immune signaling by cytosolic DNA. Science (2013) 339(6121):826–30. doi: 10.1126/science.1229963 PMC3855410 23258412

[121] Sun L Wu J Du F Chen X Chen ZJ. Cyclic GMP-AMP synthase is a cytosolic DNA sensor that activates the type I interferon pathway. Science (2013) 339(6121):786–91. doi: 10.1126/science.1232458 PMC3863629 23258413

[122] Ablasser A Goldeck M Cavlar T Deimling T Witte G Rohl I. cGAS produces a 2'-5'-linked cyclic dinucleotide second messenger that activates STING. Nature (2013) 498(7454):380–4. doi: 10.1038/nature12306 PMC4143541 23722158

[123] Gao P Ascano M Wu Y Barchet W Gaffney BL Zillinger T. Cyclic [G(2',5')pA(3',5')p] is the metazoan second messenger produced by DNA-activated cyclic GMP-AMP synthase. Cell (2013) 153(5):1094–107. doi: 10.1016/j.cell.2013.04.046 PMC4382009 23647843

[124] Zhang X Shi H Wu J Zhang X Sun L Chen C. Cyclic GMP-AMP containing mixed phosphodiester linkages is an endogenous high-affinity ligand for STING. Mol Cell (2013) 51(2):226–35. doi: 10.1016/j.molcel.2013.05.022 PMC3808999 23747010

[125] Diner EJ Burdette DL Wilson SC Monroe KM Kellenberger CA Hyodo M. The innate immune DNA sensor cGAS produces a noncanonical cyclic dinucleotide that activates human STING. Cell Rep (2013) 3(5):1355–61. doi: 10.1016/j.celrep.2013.05.009 PMC3706192 23707065

[126] Zhang C Shang G Gui X Zhang X Bai XC Chen ZJ. Structural basis of STING binding with and phosphorylation by TBK1. Nature (2019) 567(7748):394–8. doi: 10.1038/s41586-019-1000-2 PMC6862768 30842653

[127] Storek KM Gertsvolf NA Ohlson MB Monack DM. cGAS and Ifi204 cooperate to produce type I IFNs in response to francisella infection. J Immunol (2015) 194(7):3236–45. doi: 10.4049/jimmunol.1402764 PMC4367159 25710914

[128] Collins AC Cai H Li T Franco LH Li XD Nair VR. Cyclic GMP-AMP synthase is an innate immune DNA sensor for mycobacterium tuberculosis. Cell Host Microbe (2015) 17(6):820–8. doi: 10.1016/j.chom.2015.05.005 PMC4499468 26048137

[129] Gao D Wu J Wu YT Du F Aroh C Yan N. Cyclic GMP-AMP synthase is an innate immune sensor of HIV and other retroviruses. Science (2013) 341(6148):903–6. doi: 10.1126/science.1240933 PMC3860819 23929945

[130] Watson RO Bell SL MacDuff DA Kimmey JM Diner EJ Olivas J. The cytosolic sensor cGAS detects mycobacterium tuberculosis DNA to induce type I interferons and activate autophagy. Cell Host Microbe (2015) 17(6):811–9. doi: 10.1016/j.chom.2015.05.004 PMC4466081 26048136

[131] Schoggins JW MacDuff DA Imanaka N Gainey MD Shrestha B Eitson JL. Pan-viral specificity of IFN-induced genes reveals new roles for cGAS in innate immunity. Nature (2014) 505(7485):691–5. doi: 10.1038/nature12862 PMC4077721 24284630

[132] Motwani M Pesiridis S Fitzgerald KA. DNA Sensing by the cGAS-STING pathway in health and disease. Nat Rev Genet (2019) 20(11):657–74. doi: 10.1038/s41576-019-0151-1 31358977

[133] Decout A Katz JD Venkatraman S Ablasser A. The cGAS-STING pathway as a therapeutic target in inflammatory diseases. Nat Rev Immunol (2021) 21(9):548–69. doi: 10.1038/s41577-021-00524-z PMC8029610 33833439

[134] West AP Shadel GS. Mitochondrial DNA in innate immune responses and inflammatory pathology. Nat Rev Immunol (2017) 17(6):363–75. doi: 10.1038/nri.2017.21 PMC7289178 28393922

[135] Kwon J Bakhoum SF. The cytosolic DNA-sensing cGAS-STING pathway in cancer. Cancer Discovery (2020) 10(1):26–39. doi: 10.1158/2159-8290.CD-19-0761 31852718 PMC7151642

[136] Meng F Yu Z Zhang D Chen S Guan H Zhou R. Induced phase separation of mutant NF2 imprisons the cGAS-STING machinery to abrogate antitumor immunity. Mol Cell (2021) 81(20):4147–64.e7. doi: 10.1016/j.molcel.2021.07.040 34453890

[137] Wu S Zhang Q Zhang F Meng F Liu S Zhou R. HER2 recruits AKT1 to disrupt STING signalling and suppress antiviral defence and antitumour immunity. Nat Cell Biol (2019) 21(8):1027–40. doi: 10.1038/s41556-019-0352-z 31332347

[138] Paul BD Snyder SH Bohr VA. Signaling by cGAS-STING in neurodegeneration, neuroinflammation, and aging. Trends Neurosci (2021) 44(2):83–96. doi: 10.1016/j.tins.2020.10.008 33187730 PMC8662531

[139] Chen C Xu P. Cellular functions of cGAS-STING signaling. Trends Cell Biol (2022). doi: 10.1016/j.tcb.2022.11.001 36437149

[140] Dan Zhang YL Zhu Y Zhang Q Guan H Liu S Chen S. A non-canonical cGAS-STING-PERK pathway facilitates the translational program critical for senescence and organ fibrosis. Nat Cell Biol (2022) 24(5):766–82. doi: 10.1038/s41556-022-00894-z 35501370

[141] Zhang Q Liu S Zhang CS Wu Q Yu X Zhou R. AMPK directly phosphorylates TBK1 to integrate glucose sensing into innate immunity. Mol Cell (2022) 82(23):4519–36.e7. doi: 10.1016/j.molcel.2022.10.026 36384137

[142] Moriyama M Koshiba T Ichinohe T. Influenza a virus M2 protein triggers mitochondrial DNA-mediated antiviral immune responses. Nat Commun (2019) 10(1):4624. doi: 10.1038/s41467-019-12632-5 31604929 PMC6789137

[143] Corcoran JA Saffran HA Duguay BA Smiley JR. Herpes simplex virus UL12.5 targets mitochondria through a mitochondrial localization sequence proximal to the n terminus. J Virol (2009) 83(6):2601–10. doi: 10.1128/JVI.02087-08 PMC2648271 19129438

[144] Saffran HA Pare JM Corcoran JA Weller SK Smiley JR. Herpes simplex virus eliminates host mitochondrial DNA. EMBO Rep (2007) 8(2):188–93. doi: 10.1038/sj.embor.7400878 PMC1796774 17186027

[145] West AP Khoury-Hanold W Staron M Tal MC Pineda CM Lang SM. Mitochondrial DNA stress primes the antiviral innate immune response. Nature (2015) 520(7548):553–7. doi: 10.1038/nature14156 PMC4409480 25642965

[146] Duguay BA Saffran HA Ponomarev A Duley SA Eaton HE Smiley JR. Elimination of mitochondrial DNA is not required for herpes simplex virus 1 replication. J Virol (2014) 88(5):2967–76. doi: 10.1128/JVI.03129-13 PMC3958086 24371054

[147] Sun B Sundstrom KB Chew JJ Bist P Gan ES Tan HC. Dengue virus activates cGAS through the release of mitochondrial DNA. Sci Rep (2017) 7(1):3594. doi: 10.1038/s41598-017-03932-1 28620207 PMC5472572

[148] Aguirre S Luthra P Sanchez-Aparicio MT Maestre AM Patel J Lamothe F. Dengue virus NS2B protein targets cGAS for degradation and prevents mitochondrial DNA sensing during infection. Nat Microbiol (2017) 2:17037. doi: 10.1038/nmicrobiol.2017.37 28346446 PMC7457382

[149] Schoggins JW MacDuff DA Imanaka N Gainey MD Shrestha B Eitson JL. Corrigendum: pan-viral specificity of IFN-induced genes reveals new roles for cGAS in innate immunity. Nature (2015) 525(7567):144. doi: 10.1038/nature14555 PMC8323779 26153856

[150] Lai JH Wang MY Huang CY Wu CH Hung LF Yang CY. Infection with the dengue RNA virus activates TLR9 signaling in human dendritic cells. EMBO Rep (2018) 19(8):e46182. doi: 10.15252/embr.201846182 29880709 PMC6073071

[151] Wassermann R Gulen MF Sala C Perin SG Lou Y Rybniker J. Mycobacterium tuberculosis differentially activates cGAS- and inflammasome-dependent intracellular immune responses through ESX-1. Cell Host Microbe (2015) 17(6):799–810. doi: 10.1016/j.chom.2015.05.003 26048138

[152] Wiens KE Ernst JD. The mechanism for type I interferon induction by mycobacterium tuberculosis is bacterial strain-dependent. PloS Pathog (2016) 12(8):e1005809. doi: 10.1371/journal.ppat.1005809 27500737 PMC4976988

[153] Aarreberg LD Esser-Nobis K Driscoll C Shuvarikov A Roby JA Gale M Jr. Interleukin-1beta induces mtDNA release to activate innate immune signaling *via* cGAS-STING. Mol Cell (2019) 74(4):801–15.e6. doi: 10.1016/j.molcel.2019.02.038 30952515 PMC6596306

[154] Parisi MA Clayton DA. Similarity of human mitochondrial transcription factor 1 to high mobility group proteins. Science (1991) 252(5008):965–9. doi: 10.1126/science.2035027 2035027

[155] Chen L Dong J Liao S Wang S Wu Z Zuo M. Loss of Sam50 in hepatocytes induces cardiolipin-dependent mitochondrial membrane remodeling to trigger mtDNA release and liver injury. Hepatology (2022) 76(5):1389–408. doi: 10.1002/hep.32471 35313046

[156] Wu Z Oeck S West AP Mangalhara KC Sainz AG Newman LE. Mitochondrial DNA stress signalling protects the nuclear genome. Nat Metab (2019) 1(12):1209–18. doi: 10.1038/s42255-019-0150-8 PMC7213273 32395698

[157] Sprenger HG MacVicar T Bahat A Fiedler KU Hermans S Ehrentraut D. Cellular pyrimidine imbalance triggers mitochondrial DNA-dependent innate immunity. Nat Metab (2021) 3(5):636–50. doi: 10.1038/s42255-021-00385-9 PMC8144018 33903774

[158] Zhang Q Chen C Xia B Xu P. Chemical regulation of the cGAS-STING pathway. Curr Opin Chem Biol (2022) 69:102170. doi: 10.1016/j.cbpa.2022.102170 35753220

[159] Nakahira K Haspel JA Rathinam VA Lee SJ Dolinay T Lam HC. Autophagy proteins regulate innate immune responses by inhibiting the release of mitochondrial DNA mediated by the NALP3 inflammasome. Nat Immunol (2011) 12(3):222–30. doi: 10.1038/ni.1980 PMC3079381 21151103

[160] Zhong Z Liang S Sanchez-Lopez E He F Shalapour S Lin XJ. New mitochondrial DNA synthesis enables NLRP3 inflammasome activation. Nature (2018) 560(7717):198–203. doi: 10.1038/s41586-018-0372-z 30046112 PMC6329306

[161] Tumurkhuu G Shimada K Dagvadorj J Crother TR Zhang W Luthringer D. Ogg1-dependent DNA repair regulates NLRP3 inflammasome and prevents atherosclerosis. Circ Res (2016) 119(6):e76–90. doi: 10.1161/CIRCRESAHA.116.308362 PMC5010464 27384322

[162] Shimada K Crother TR Karlin J Dagvadorj J Chiba N Chen S. Oxidized mitochondrial DNA activates the NLRP3 inflammasome during apoptosis. Immunity (2012) 36(3):401–14. doi: 10.1016/j.immuni.2012.01.009 PMC3312986 22342844

[163] Yu J Nagasu H Murakami T Hoang H Broderick L Hoffman HM. Inflammasome activation leads to caspase-1-dependent mitochondrial damage and block of mitophagy. Proc Natl Acad Sci U S A. (2014) 111(43):15514–9. doi: 10.1073/pnas.1414859111 PMC4217429 25313054

[164] Bronner DN Abuaita BH Chen X Fitzgerald KA Nunez G He Y. Endoplasmic reticulum stress activates the inflammasome *via* NLRP3- and caspase-2-Driven mitochondrial damage. Immunity (2015) 43(3):451–62. doi: 10.1016/j.immuni.2015.08.008 PMC4582788 26341399

[165] Fitzgerald KA Kagan JC. Toll-like receptors and the control of immunity. Cell (2020) 180(6):1044–66. doi: 10.1016/j.cell.2020.02.041 PMC9358771 32164908

[166] Zhang Q Raoof M Chen Y Sumi Y Sursal T Junger W. Circulating mitochondrial DAMPs cause inflammatory responses to injury. Nature (2010) 464(7285):104–7. doi: 10.1038/nature08780 PMC2843437 20203610

[167] Zhang Q Itagaki K Hauser CJ. Mitochondrial DNA is released by shock and activates neutrophils *via* p38 map kinase. Shock (2010) 34(1):55–9. doi: 10.1097/SHK.0b013e3181cd8c08 19997055

[168] Zhang Z Meng P Han Y Shen C Li B Hakim MA. Mitochondrial DNA-LL-37 complex promotes atherosclerosis by escaping from autophagic recognition. Immunity (2015) 43(6):1137–47. doi: 10.1016/j.immuni.2015.10.018 26680206

[169] Julian MW Shao G Bao S Knoell DL Papenfuss TL VanGundy ZC. Mitochondrial transcription factor a serves as a danger signal by augmenting plasmacytoid dendritic cell responses to DNA. J Immunol (2012) 189(1):433–43. doi: 10.4049/jimmunol.1101375 PMC3381894 22675199

[170] Julian MW Shao G Vangundy ZC Papenfuss TL Crouser ED. Mitochondrial transcription factor a, an endogenous danger signal, promotes TNFalpha release *via* RAGE- and TLR9-responsive plasmacytoid dendritic cells. PloS One (2013) 8(8):e72354. doi: 10.1371/journal.pone.0072354 23951313 PMC3741150

[171] Tezze C Romanello V Desbats MA Fadini GP Albiero M Favaro G. Age-Associated Loss of OPA1 in Muscle Impacts Muscle Mass, Metabolic Homeostasis, Systemic Inflammation, and Epithelial Senescence. Cell Metab (2017) 25(6):1374–89.e6 10.1016/j.cmet.2017.04.021 PMC5462533 28552492

[172] Pereira RO Tadinada SM Zasadny FM Oliveira KJ Pires KMP Olvera A. OPA1 deficiency promotes secretion of FGF21 from muscle that prevents obesity and insulin resistance. EMBO J (2017) 36(14):2126–45. doi: 10.15252/embj.201696179 PMC5510002 28607005

[173] Rodriguez-Nuevo A Diaz-Ramos A Noguera E Diaz-Saez F Duran X Munoz JP. Mitochondrial DNA and TLR9 drive muscle inflammation upon Opa1 deficiency. EMBO J (2018) 37(10):e96553. doi: 10.15252/embj.201796553 29632021 PMC5978453

[174] Ding Z Liu S Wang X Khaidakov M Dai Y Mehta JL. Oxidant stress in mitochondrial DNA damage, autophagy and inflammation in atherosclerosis. Sci Rep (2013) 3:1077. doi: 10.1038/srep01077 23326634 PMC3546319

[175] McCarthy CG Wenceslau CF Goulopoulou S Ogbi S Baban B Sullivan JC. Circulating mitochondrial DNA and toll-like receptor 9 are associated with vascular dysfunction in spontaneously hypertensive rats. Cardiovasc Res (2015) 107(1):119–30. doi: 10.1093/cvr/cvv137 PMC4560046 25910936

[176] Marques PE Amaral SS Pires DA Nogueira LL Soriani FM Lima BH. Chemokines and mitochondrial products activate neutrophils to amplify organ injury during mouse acute liver failure. Hepatology (2012) 56(5):1971–82. doi: 10.1002/hep.25801 22532075

[177] Garcia-Martinez I Santoro N Chen Y Hoque R Ouyang X Caprio S. Hepatocyte mitochondrial DNA drives nonalcoholic steatohepatitis by activation of TLR9. J Clin Invest. (2016) 126(3):859–64. doi: 10.1172/JCI83885 PMC4767345 26808498

[178] Lood C Blanco LP Purmalek MM Carmona-Rivera C De Ravin SS Smith CK. Neutrophil extracellular traps enriched in oxidized mitochondrial DNA are interferogenic and contribute to lupus-like disease. Nat Med (2016) 22(2):146–53. doi: 10.1038/nm.4027 PMC4742415 26779811

[179] Caielli S Athale S Domic B Murat E Chandra M Banchereau R. Oxidized mitochondrial nucleoids released by neutrophils drive type I interferon production in human lupus. J Exp Med (2016) 213(5):697–713. doi: 10.1084/jem.20151876 27091841 PMC4854735

[180] Papayannopoulos V. Neutrophil extracellular traps in immunity and disease. Nat Rev Immunol (2018) 18(2):134–47. doi: 10.1038/nri.2017.105 28990587

[181] Brinkmann V Reichard U Goosmann C Fauler B Uhlemann Y Weiss DS. Neutrophil extracellular traps kill bacteria. Science (2004) 303(5663):1532–5. doi: 10.1126/science.1092385 15001782

[182] Shin JH Yang JY Jeon BY Yoon YJ Cho SN Kang YH. (1)H NMR-based metabolomic profiling in mice infected with mycobacterium tuberculosis. J Proteome Res (2011) 10(5):2238–47. doi: 10.1021/pr101054m 21452902

[183] Gkirtzimanaki K Kabrani E Nikoleri D Polyzos A Blanas A Sidiropoulos P. IFNalpha impairs autophagic degradation of mtDNA promoting autoreactivity of SLE monocytes in a STING-dependent fashion. Cell Rep (2018) 25(4):921–33 e5. doi: 10.1016/j.celrep.2018.09.001 30355498 PMC6218203

[184] Yousefi S Gold JA Andina N Lee JJ Kelly AM Kozlowski E. Catapult-like release of mitochondrial DNA by eosinophils contributes to antibacterial defense. Nat Med (2008) 14(9):949–53. doi: 10.1038/nm.1855 18690244

[185] Ingelsson B Soderberg D Strid T Soderberg A Bergh AC Loitto V. Lymphocytes eject interferogenic mitochondrial DNA webs in response to CpG and non-CpG oligodeoxynucleotides of class c. Proc Natl Acad Sci U S A. (2018) 115(3):E478–E87. doi: 10.1073/pnas.1711950115 PMC5776968 29295921

[186] Grochowska J Czerwinska J Borowski LS Szczesny RJ. Mitochondrial RNA, a new trigger of the innate immune system. Wiley Interdiscip Rev RNA. (2022) 13(3):e1690. doi: 10.1002/wrna.1690 34498404

[187] Ojala D Montoya J Attardi G. tRNA punctuation model of RNA processing in human mitochondria. Nature (1981) 290(5806):470–4. doi: 10.1038/290470a0 7219536

[188] Borowski LS Dziembowski A Hejnowicz MS Stepien PP Szczesny RJ. Human mitochondrial RNA decay mediated by PNPase-hSuv3 complex takes place in distinct foci. Nucleic Acids Res (2013) 41(2):1223–40. doi: 10.1093/nar/gks1130 PMC3553951 23221631

[189] Szczesny RJ Borowski LS Brzezniak LK Dmochowska A Gewartowski K Bartnik E. Human mitochondrial RNA turnover caught in flagranti: involvement of hSuv3p helicase in RNA surveillance. Nucleic Acids Res (2010) 38(1):279–98. doi: 10.1093/nar/gkp903 PMC2800237 19864255

[190] Dhir A Dhir S Borowski LS Jimenez L Teitell M Rotig A. Mitochondrial double-stranded RNA triggers antiviral signalling in humans. Nature (2018) 560(7717):238–42. doi: 10.1038/s41586-018-0363-0 PMC6570621 30046113

[191] Rius R Van Bergen NJ Compton AG Riley LG Kava MP Balasubramaniam S. Clinical spectrum and functional consequences associated with bi-allelic pathogenic PNPT1 variants. J Clin Med (2019) 8(11):2020. doi: 10.3390/jcm8112020 31752325 PMC6912252

[192] Kim Y Park J Kim S Kim M Kang MG Kwak C. PKR senses nuclear and mitochondrial signals by interacting with endogenous double-stranded RNAs. Mol Cell (2018) 71(6):1051–63.e6. doi: 10.1016/j.molcel.2018.07.029 30174290

[193] Forrester SJ Kikuchi DS Hernandes MS Xu Q Griendling KK. Reactive oxygen species in metabolic and inflammatory signaling. Circ Res (2018) 122(6):877–902. doi: 10.1161/CIRCRESAHA.117.311401 29700084 PMC5926825

[194] Silwal P Kim JK Kim YJ Jo EK. Mitochondrial reactive oxygen species: double-edged weapon in host defense and pathological inflammation during infection. Front Immunol (2020) 11:1649. doi: 10.3389/fimmu.2020.01649 32922385 PMC7457135

[195] Zhao Y Sun X Nie X Sun L Tang TS Chen D. COX5B regulates MAVS-mediated antiviral signaling through interaction with ATG5 and repressing ROS production. PloS Pathog (2012) 8(12):e1003086. doi: 10.1371/journal.ppat.1003086 23308066 PMC3534373

[196] Wang R Zhu Y Lin X Ren C Zhao J Wang F. Influenza M2 protein regulates MAVS-mediated signaling pathway through interacting with MAVS and increasing ROS production. Autophagy (2019) 15(7):1163–81. doi: 10.1080/15548627.2019.1580089 PMC6613841 30741586

[197] Kozaki T Komano J Kanbayashi D Takahama M Misawa T Satoh T. Mitochondrial damage elicits a TCDD-inducible poly(ADP-ribose) polymerase-mediated antiviral response. Proc Natl Acad Sci U S A. (2017) 114(10):2681–6. doi: 10.1073/pnas.1621508114 PMC5347618 28213497

[198] Gross CJ Mishra R Schneider KS Medard G Wettmarshausen J Dittlein DC. K(+) efflux-independent NLRP3 inflammasome activation by small molecules targeting mitochondria. Immunity (2016) 45(4):761–73. doi: 10.1016/j.immuni.2016.08.010 27692612

[199] Lemasters JJ Theruvath TP Zhong Z Nieminen AL. Mitochondrial calcium and the permeability transition in cell death. Biochim Biophys Acta (2009) 1787(11):1395–401. doi: 10.1016/j.bbabio.2009.06.009 PMC2730424 19576166

[200] Bonora M Giorgi C Pinton P. Molecular mechanisms and consequences of mitochondrial permeability transition. Nat Rev Mol Cell Biol (2022) 23(4):266–85. doi: 10.1038/s41580-021-00433-y 34880425

[201] Bai D Du J Bu X Cao W Sun T Zhao J. ALDOA maintains NLRP3 inflammasome activation by controlling AMPK activation. Autophagy (2022) 18(7):1673–93. doi: 10.1080/15548627.2021.1997051 PMC9298449 34821530

[202] Vorobjeva N Galkin I Pletjushkina O Golyshev S Zinovkin R Prikhodko A. Mitochondrial permeability transition pore is involved in oxidative burst and NETosis of human neutrophils. Biochim Biophys Acta Mol Basis Dis (2020) 1866(5):165664. doi: 10.1016/j.bbadis.2020.165664 31926265

[203] Rousset S Emre Y Join-Lambert O Hurtaud C Ricquier D Cassard-Doulcier AM. The uncoupling protein 2 modulates the cytokine balance in innate immunity. Cytokine (2006) 35(3-4):135–42. doi: 10.1016/j.cyto.2006.07.012 16971137

[204] Vorobjeva N Prikhodko A Galkin I Pletjushkina O Zinovkin R Sud'ina G. Mitochondrial reactive oxygen species are involved in chemoattractant-induced oxidative burst and degranulation of human neutrophils in vitro. Eur J Cell Biol (2017) 96(3):254–65. doi: 10.1016/j.ejcb.2017.03.003 28325500

[205] Lee SY Park SH Lee SW Lee SH Son MK Choi YH. Synoviocyte apoptosis may differentiate responder and non-responder patients to methotrexate treatment in rheumatoid arthritis. Arch Pharm Res (2014) 37(10):1286–94. doi: 10.1007/s12272-014-0365-x 24988987

[206] West AP Brodsky IE Rahner C Woo DK Erdjument-Bromage H Tempst P. TLR signalling augments macrophage bactericidal activity through mitochondrial ROS. Nature (2011) 472(7344):476–80. doi: 10.1038/nature09973 PMC3460538 21525932

[207] Carneiro FRG Lepelley A Seeley JJ Hayden MS Ghosh S. An essential role for ECSIT in mitochondrial complex I assembly and mitophagy in macrophages. Cell Rep (2018) 22(10):2654–66. doi: 10.1016/j.celrep.2018.02.051 PMC5909989 29514094

[208] Sonoda J Laganiere J Mehl IR Barish GD Chong LW Li X. Nuclear receptor ERR alpha and coactivator PGC-1 beta are effectors of IFN-gamma-induced host defense. Genes Dev (2007) 21(15):1909–20. doi: 10.1101/gad.1553007 PMC1935029 17671090

[209] Bulua AC Simon A Maddipati R Pelletier M Park H Kim KY. Mitochondrial reactive oxygen species promote production of proinflammatory cytokines and are elevated in TNFR1-associated periodic syndrome (TRAPS). J Exp Med (2011) 208(3):519–33. doi: 10.1084/jem.20102049 PMC3058571 21282379

[210] Hwang AB Ryu EA Artan M Chang HW Kabir MH Nam HJ. Feedback regulation *via* AMPK and HIF-1 mediates ROS-dependent longevity in caenorhabditis elegans. Proc Natl Acad Sci U S A. (2014) 111(42):E4458–67. doi: 10.1073/pnas.1411199111 PMC4210294 25288734

[211] Kwon S Kim EJE Lee SV. Mitochondria-mediated defense mechanisms against pathogens in caenorhabditis elegans. BMB Rep (2018) 51(6):274–9. doi: 10.5483/BMBRep.2018.51.6.111 PMC6033066 29764564

[212] Lee MKS Al-Sharea A Shihata WA Bertuzzo Veiga C Cooney OD Fleetwood AJ. Glycolysis is required for LPS-induced activation and adhesion of human CD14(+)CD16(-) monocytes. Front Immunol (2019) 10:2054. doi: 10.3389/fimmu.2019.02054 31555280 PMC6742687

[213] Goncalves SM Duarte-Oliveira C Campos CF Aimanianda V Ter Horst R Leite L. Phagosomal removal of fungal melanin reprograms macrophage metabolism to promote antifungal immunity. Nat Commun (2020) 11(1):2282. doi: 10.1038/s41467-020-16120-z 32385235 PMC7210971

[214] Rojas Marquez JD Ana Y Baigorri RE Stempin CC Cerban FM. Mammalian target of rapamycin inhibition in trypanosoma cruzi-infected macrophages leads to an intracellular profile that is detrimental for infection. Front Immunol (2018) 9:313. doi: 10.3389/fimmu.2018.00313 29515594 PMC5826284

[215] Weindel CG Martinez EL Zhao X Mabry CJ Bell SL Vail KJ. Mitochondrial ROS promotes susceptibility to infection *via* gasdermin d-mediated necroptosis. Cell (2022) 185(17):3214–31.e23. doi: 10.1016/j.cell.2022.06.038 35907404 PMC9531054

[216] Zhang W Wang G Xu ZG Tu H Hu F Dai J. Lactate is a natural suppressor of RLR signaling by targeting MAVS. Cell (2019) 178(1):176–89.e15. doi: 10.1016/j.cell.2019.05.003 31155231 PMC6625351

[217] Chao CC Gutierrez-Vazquez C Rothhammer V Mayo L Wheeler MA Tjon EC. Metabolic control of astrocyte pathogenic activity *via* cPLA2-MAVS. Cell (2019) 179(7):1483–98.e22. doi: 10.1016/j.cell.2019.11.016 31813625 PMC6936326

[218] Wolf AJ Reyes CN Liang W Becker C Shimada K Wheeler ML. Hexokinase is an innate immune receptor for the detection of bacterial peptidoglycan. Cell (2016) 166(3):624–36. doi: 10.1016/j.cell.2016.05.076 PMC5534359 27374331

[219] Sanman LE Qian Y Eisele NA Ng TM van der Linden WA Monack DM. Disruption of glycolytic flux is a signal for inflammasome signaling and pyroptotic cell death. Elife (2016) 5:e13663. doi: 10.7554/eLife.13663 27011353 PMC4846378

[220] Wen H Gris D Lei Y Jha S Zhang L Huang MT. Fatty acid-induced NLRP3-ASC inflammasome activation interferes with insulin signaling. Nat Immunol (2011) 12(5):408–15. doi: 10.1038/ni.2022 PMC4090391 21478880

[221] Moon JS Nakahira K Chung KP DeNicola GM Koo MJ Pabon MA. NOX4-dependent fatty acid oxidation promotes NLRP3 inflammasome activation in macrophages. Nat Med (2016) 22(9):1002–12. doi: 10.1038/nm.4153 PMC5204248 27455510

[222] Moon JS Lee S Park MA Siempos II Haslip M PJ L. UCP2-induced fatty acid synthase promotes NLRP3 inflammasome activation during sepsis. J Clin Invest. (2015) 125(2):665–80. doi: 10.1172/JCI78253 PMC4319445 25574840

[223] Li XN Song J Zhang L LeMaire SA Hou X Zhang C. Activation of the AMPK-FOXO3 pathway reduces fatty acid-induced increase in intracellular reactive oxygen species by upregulating thioredoxin. Diabetes (2009) 58(10):2246–57. doi: 10.2337/db08-1512 PMC2750236 19592618

[224] Youm YH Nguyen KY Grant RW Goldberg EL Bodogai M Kim D. The ketone metabolite beta-hydroxybutyrate blocks NLRP3 inflammasome-mediated inflammatory disease. Nat Med (2015) 21(3):263–9. doi: 10.1038/nm.3804 PMC4352123 25686106

[225] Deng Y Xie M Li Q Xu X Ou W Zhang Y. Targeting mitochondria-inflammation circuit by beta-hydroxybutyrate mitigates HFpEF. Circ Res (2021) 128(2):232–45. doi: 10.1161/CIRCRESAHA.120.317933 33176578

[226] Truax AD Chen L Tam JW Cheng N Guo H Koblansky AA. The inhibitory innate immune sensor NLRP12 maintains a threshold against obesity by regulating gut microbiota homeostasis. Cell Host Microbe (2018) 24(3):364–78.e6. doi: 10.1016/j.chom.2018.08.009 30212649 PMC6161752

[227] Willenborg S Sanin DE Jais A Ding X Ulas T Nüchel J. Mitochondrial metabolism coordinates stage-specific repair processes in macrophages during wound healing. Cell Metab (2021) 33(12):2398–414.e9. doi: 10.1016/j.cmet.2021.10.004 34715039

[228] Wang Y Li N Zhang X Horng T. Mitochondrial metabolism regulates macrophage biology. J Biol Chem (2021) 297(1):100904. doi: 10.1016/j.jbc.2021.100904 34157289 PMC8294576

[229] Huang SC Everts B Ivanova Y O'Sullivan D Nascimento M Smith AM. Cell-intrinsic lysosomal lipolysis is essential for alternative activation of macrophages. Nat Immunol (2014) 15(9):846–55. doi: 10.1038/ni.2956 PMC4139419 25086775

[230] Dong T Chen X Xu H Song Y Wang H Gao Y. Mitochondrial metabolism mediated macrophage polarization in chronic lung diseases. Pharmacol Ther (2022) 239:108208. doi: 10.1016/j.pharmthera.2022.108208 35569553

[231] Martinez-Reyes I Chandel NS. Mitochondrial TCA cycle metabolites control physiology and disease. Nat Commun (2020) 11(1):102. doi: 10.1038/s41467-019-13668-3 31900386 PMC6941980

[232] Lee JV Carrer A Shah S Snyder NW Wei S Venneti S. Akt-dependent metabolic reprogramming regulates tumor cell histone acetylation. Cell Metab (2014) 20(2):306–19. doi: 10.1016/j.cmet.2014.06.004 PMC4151270 24998913

[233] McDonnell E Crown SB Fox DB Kitir B Ilkayeva OR Olsen CA. Lipids reprogram metabolism to become a major carbon source for histone acetylation. Cell Rep (2016) 17(6):1463–72. doi: 10.1016/j.celrep.2016.10.012 PMC5123807 27806287

[234] Martinez-Reyes I Diebold LP Kong H Schieber M Huang H Hensley CT. TCA cycle and mitochondrial membrane potential are necessary for diverse biological functions. Mol Cell (2016) 61(2):199–209. doi: 10.1016/j.molcel.2015.12.002 26725009 PMC4724312

[235] Wellen KE Hatzivassiliou G Sachdeva UM Bui TV Cross JR Thompson CB. ATP-citrate lyase links cellular metabolism to histone acetylation. Science (2009) 324(5930):1076–80. doi: 10.1126/science.1164097 PMC2746744 19461003

[236] Infantino V Iacobazzi V Palmieri F Menga A. ATP-citrate lyase is essential for macrophage inflammatory response. Biochem Biophys Res Commun (2013) 440(1):105–11. doi: 10.1016/j.bbrc.2013.09.037 24051091

[237] Covarrubias AJ Aksoylar HI Yu J Snyder NW Worth AJ Iyer SS. Akt-mTORC1 signaling regulates acly to integrate metabolic input to control of macrophage activation. Elife (2016) 5:e11612. doi: 10.7554/eLife.11612 26894960 PMC4769166

[238] Michelucci A Cordes T Ghelfi J Pailot A Reiling N Goldmann O. Immune-responsive gene 1 protein links metabolism to immunity by catalyzing itaconic acid production. Proc Natl Acad Sci U S A. (2013) 110(19):7820–5. doi: 10.1073/pnas.1218599110 PMC3651434 23610393

[239] Degrandi D Hoffmann R Beuter-Gunia C Pfeffer K. The proinflammatory cytokine-induced IRG1 protein associates with mitochondria. J Interferon Cytokine Res (2009) 29(1):55–67. doi: 10.1089/jir.2008.0013 19014335

[240] Sugimoto M Sakagami H Yokote Y Onuma H Kaneko M Mori M. Non-targeted metabolite profiling in activated macrophage secretion. Metabolomics (2012) 8(4):624–33. doi: 10.1007/s11306-011-0353-9

[241] Lampropoulou V Sergushichev A Bambouskova M Nair S Vincent EE Loginicheva E. Itaconate links inhibition of succinate dehydrogenase with macrophage metabolic remodeling and regulation of inflammation. Cell Metab (2016) 24(1):158–66. doi: 10.1016/j.cmet.2016.06.004 PMC5108454 27374498

[242] Liu PS Wang H Li X Chao T Teav T Christen S. Alpha-ketoglutarate orchestrates macrophage activation through metabolic and epigenetic reprogramming. Nat Immunol (2017) 18(9):985–94. doi: 10.1038/ni.3796 28714978

[243] Tannahill GM Curtis AM Adamik J Palsson-McDermott EM McGettrick AF Goel G. Succinate is an inflammatory signal that induces IL-1beta through HIF-1alpha. Nature (2013) 496(7444):238–42. doi: 10.1038/nature11986 PMC4031686 23535595

[244] Mills EL Kelly B Logan A Costa ASH Varma M Bryant CE. Succinate dehydrogenase supports metabolic repurposing of mitochondria to drive inflammatory macrophages. Cell (2016) 167(2):457–70.e13. doi: 10.1016/j.cell.2016.08.064 27667687 PMC5863951

[245] Jha AK Huang SC Sergushichev A Lampropoulou V Ivanova Y Loginicheva E. Network integration of parallel metabolic and transcriptional data reveals metabolic modules that regulate macrophage polarization. Immunity (2015) 42(3):419–30. doi: 10.1016/j.immuni.2015.02.005 25786174

[246] Arts RJ Novakovic B Ter Horst R Carvalho A Bekkering S Lachmandas E. Glutaminolysis and fumarate accumulation integrate immunometabolic and epigenetic programs in trained immunity. Cell Metab (2016) 24(6):807–19. doi: 10.1016/j.cmet.2016.10.008 PMC5742541 27866838

